# Optimizing Interfacial Energetics for Conjugated Polyelectrolyte Electron Injection Layers in High Efficiency and Fast Responding Polymer Light Emitting Diodes

**DOI:** 10.1021/acsami.2c05640

**Published:** 2022-05-18

**Authors:** Iain Hamilton, Minwon Suh, Jim Bailey, Donal D. C. Bradley, Ji-Seon Kim

**Affiliations:** † Department of Physics and Centre for Processable Electronics, Imperial College London, London SW7 2AZ, United Kingdom; ‡ Division of Physical Sciences and Engineering, King Abdullah University of Science and Technology (KAUST), Thuwal, 23955−6900 Saudi Arabia

**Keywords:** conjugated polyelectrolytes, electron injection layers, polymer light-emitting diodes, interfaces

## Abstract

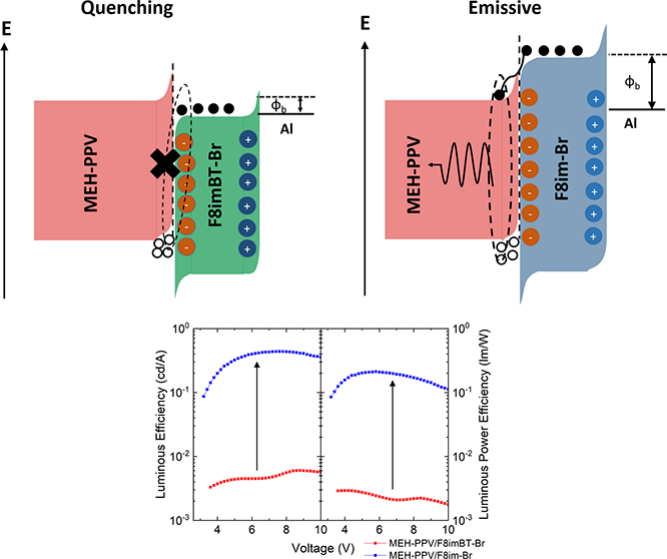

Modification of the π-conjugated backbone structure of conjugated polyelectrolytes (CPEs) for use as electron injection layers (EILs) in polymer light emitting diodes (PLEDs) has previously brought conflicted results in the literature in terms of device efficiency and turn-on response time. Herein, we determine the energetics at the CPE and the light emitting polymer (LEP) interface as a key factor for PLED device performance. By varying the conjugated backbone structure of both the LEP and CPE, we control the nature of the CPE/LEP interface in terms of optical energy gap offset, interfacial energy level offset, and location of the electron–hole recombination zone. We use a wide gap CPE with a shallow LUMO (F8im-Br) and one with a smaller gap and deeper LUMO (F8imBT-Br), in combination with three different LEPs. We find that the formation of a type II heterojunction at the CPE/LEP interfaces causes interfacial luminance quenching, which is responsible for poor efficiency in PLED devices. The effect is exacerbated with increased energy level offset from ionic rearrangement and hole accumulation occurring near the CPE/LEP interface. However, a deep CPE LUMO is found to be beneficial for fast current and luminance turn-on times of devices. This work provides important CPE molecular design rules for EIL use, offering progress toward a universal PLED-compatible CPE that can simultaneously deliver high efficiency and fast response times. In particular, engineering the LUMO position to be deep enough for fast device turn-on while avoiding the creation of a large energy level offset at the CPE/LEP interface is shown to be highly desirable.

## Introduction

Conjugated polyelectrolytes (CPEs) are a class of polymers chiefly characterized by a delocalized π-conjugated backbone with tethered pendant ionic groups. The ionic functionality allows CPEs to be dissolved in alcohol- or water-based polar solvents and makes them particularly attractive for use in multilayer organic optoelectronic devices on account of the resulting “orthogonality” to typical organic solvent deposited active layer materials and allows the realization of all-printable devices.^1−3^

Recently, CPEs have found use as interlayer materials in organic optoelectronic devices.^4−7^ In particular, thin (5–20 nm) CPE electron injection layers (EILs) have been found to greatly enhance electron injection from high workfunction electrodes into polymer light-emitting diodes (PLEDs) and improve device efficiency.^1,3,8−13^ This allows CPEs to replace traditional low workfunction metals that are both less stable and less environmentally friendly.^3^ CPEs can also reduce luminescence quenching in PLEDs such as by reducing image charge quenching from metal interfaces^14,15^ and passivating quenching states in metal oxides.^16,17^ However, CPEs themselves are poor emitters with the ions known to quench luminescence.^18^ Other sources of quenching include chemical defects of the emitting layer^19^ and solid state concentration quenching.^20^

The precise mechanism for the enhanced electron injection in CPE-based PLEDs is still unclear. Traditionally, for very thin CPE layers (<10 nm) the electric dipole from the ions induces a dipole in the metal cathode which reduces the cathode workfunction with respect to the vacuum level and allows for easier electron injection.^21^ For thicker CPE layers (typically 10–30 nm), however, it is suggested that the ions undergo a rearrangement under an applied electric field and form a layer of ions at both the metal/CPE and CPE/active layer interfaces at which the electric field falls over, allowing tunnelling from the metal into the bulk CPE.^11,22,23^ CPE interfacial layers have also been used to improve the device performance of organic photovoltaics (OPVs)^24−27^ and organic field effect transistors (OFETs).^28^ CPEs can also function as the active layers in light emitting electrochemical cells (LECs)^29^ and biosensors.^30^ Alongside the development of CPEs, other interlayer materials such as nonconjugated polyelectrolytes,^31^ neutral conjugated polymers,^31^ conductive ionenes,^32,33^ and composite organic–inorganic materials^33^ have been developed that have shown promise in optoelectronic devices.

One of the drawbacks of using CPEs as EILs in PLEDs is the aforementioned rearrangement of the counterions following application of an electric field across the device as this can significantly increase the response times in PLEDs to a few seconds, which is too slow for display purposes where fast switching is required.^11,12,34^ Previous literature has focused on modifying the size and structure of the counterions, whereby large ions (such as tetrakis(1-imidazolyl)borate (BIm_4_) anions) are exchanged for smaller halide ions (such as F^–^ ions) to improve device response. The smaller ions are less disruptive to chain packing and can be transported faster, although even with this change, response times remain too long.^9,35^ Other methods to improve device response include synthesis of zwitterionic CPEs where both cation and anion groups are tethered to the conjugated backbone.^36−38^ Blending CPEs with zinc oxide nanoparticles has also been shown to improve device response times.^16^ Modification of the conjugated backbone structure of CPEs has been another strategy but has previously produced mixed results; incorporating benzothiadiazole units into otherwise fluorene backbones has succeeded in increasing PLED device performance in some cases,^15^ while in others it has led to drastically reduced device performance, the reasons for which are poorly understood.^37,39^

This work looks to explain how varying the π-conjugated structure of CPEs affects both PLED electroluminescence (EL) turn-on times and device performance (luminous and luminous power efficiency). The influence of the CPE/cathode interface and the light emitting polymer (LEP)/CPE interface are closely examined. We study the device performance of PLEDs containing three different LEPs: F8BT, an F8BT-TFB statistical copolymer, and MEH-PPV. These LEPs were chosen due to their variation in (i) optical gap, (ii) energy level positions, and (iii) charge transport properties. This allows the key factors that determine PLED device performance to be identified. For each LEP, a wide gap, polyfluorene-based CPE (F8im-Br), a F8BT based CPE (F8imBT-Br), and a low-work-function metal (Ca) are compared as electron injection layers.

We find that a combination of hole accumulation and energy level offset at the CPE/LEP interface are key factors in explaining variations in device efficiency. Energy level misalignment causes luminescence quenching across the interface, the effect of which is exacerbated by ionic rearrangement within the CPE layer and hole accumulation, which increases the energy offset and locates the recombination zone closer to the CPE/LEP. These results give fresh insight into the mechanisms of poor CPE-based PLED device performance and suggest that the design of CPEs with (i) wide enough gaps to reduce exciton quenching and (ii) deep enough LUMOs to allow for fast electron injection can lead to a “universal” CPE that displays both highly efficient and fast responding device characteristics regardless of the LEP chosen.

## Results and Discussion

### CPE and LEP Materials Properties

**Figure 1:**
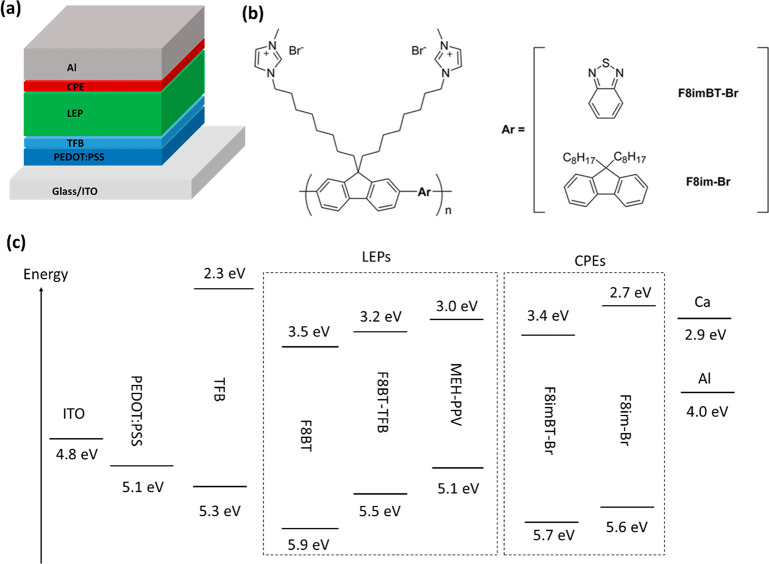
(a) Device structure of PLEDs under investigation. (b) Chemical structures of F8imBT-Br and F8im-Br. (c) Energy levels of materials used in this study. TFB energy levels are taken from ref (43). APS measurements for each LEP and CPE material can be found in Figures S2 and S3 (Supporting Information).

The PLEDs are fabricated with two different CPE structures (one based on a polyfluorene backbone, the other based on an F8BT backbone) in combination with three different LEP materials. In each case, the PLED is optimized by the inclusion of a thin layer of the hole-injection/electron-blocking interlayer poly(9,9-dioctylfluorene-*co*-N-(4-butylphenyl)-diphenylamine) (TFB).^40^ A control device using a Ca/Al cathode in place of a CPE was also fabricated. The device structure is shown in Figure 1a.

The CPE materials used in this study are (i) poly[(9,9-bis(8′-(3″-methyl-1″-imidazolium)octyl)-2,7-fluorene)-*alt*-2,7-(9,9-dioctylfluorene)] dibromide (F8im-Br), a wide gap CPE based on polyfluorene, and (ii) a fluorene-benzothiadiazole copolymer, namely poly[(9,9-bis(8′-(3″-methyl-1″-imidazolium)octyl)-2,7-fluorene)-*alt*-(benzo(2,1,3)thiadiazol-4,8-diyl) dibromide (F8imBT-Br). The chemical structures of both F8im-Br and F8imBT-Br are shown in Figure 1b, and their normalized thin film UV–vis absorption and photoluminescence spectra are presented in Figure S1.

Figure S2 shows the air photoemission spectra (APS) of F8im-Br and F8imBT-Br, from which the HOMO energy levels of the CPEs are estimated to be 5.6 and 5.7 eV, respectively. By the addition of the optical gap energies found from the UV–vis spectra, the LUMO levels are calculated to be 2.7 and 3.4 eV for F8im-Br and F8imBT-Br, respectively (see Table S1).

For each CPE material, three different LEPs are examined: F8BT, F8BT-TFB, and MEH-PPV. The π-conjugated structure of the LEP causes several factors to change, most importantly in this study (i) the optical gap, (ii) the position of the HOMO and LUMO levels, and (iii) the relative hole and electron mobilities. By altering these factors, the type of heterojunction formed at the CPE/LEP interface is modified for electron injection, electron transport, and energy transfer; the effect this has on device performance will be investigated below. The relative hole and electron mobilities help to determine the location of the recombination zone (RZ) within the LEP device.^43,42^ F8BT is primarily an electron transporting polymer,^44,45^ while F8BT-TFB has balanced charged transport.^46^ MEH-PPV is a hole transporting polymer.^47−49^ The chemical structures and energy levels (measured using APS, Figure S3) for each LEP are shown below in Table 1, while their UV–visible absorption and PL spectra are shown in Figure S4. More details on the chemical structures, charge mobilities, and optoelectronic properties of each LEP are discussed in the Supporting Information.

**Table 1:**
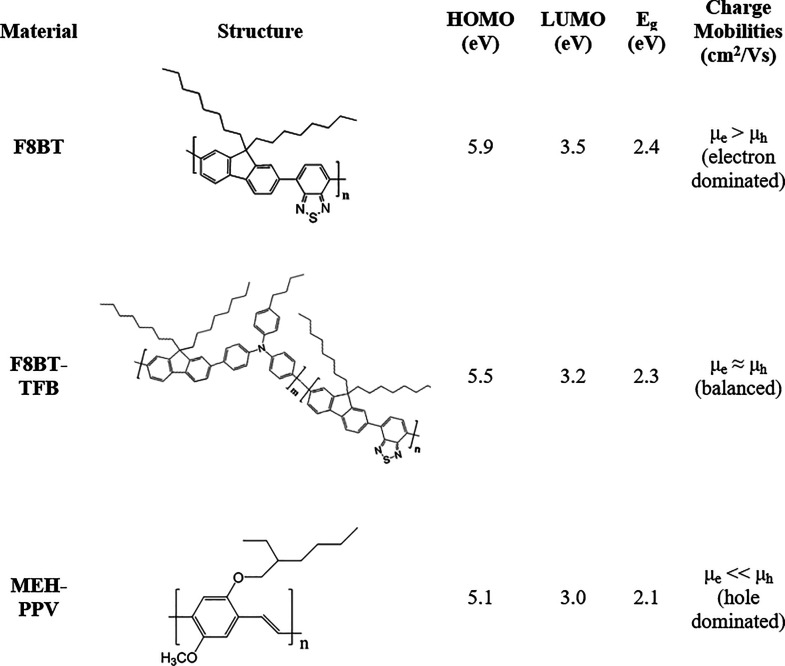
Light Emitting Polymer Chemical Structures, Estimated HOMO and LUMO Energies, and Their Relative Hole and Electron Mobilities

Atomic force microscopy (AFM) measurements were conducted on each of the LEP and CPE materials (Figure S5), all of which show smooth, featureless morphologies with low roughness values (*R*_q_ < 1 nm), implying the morphologies of the different material combinations are unlikely to impact on device performance.

### Light-Emitting Diodes

**Figure 2:**
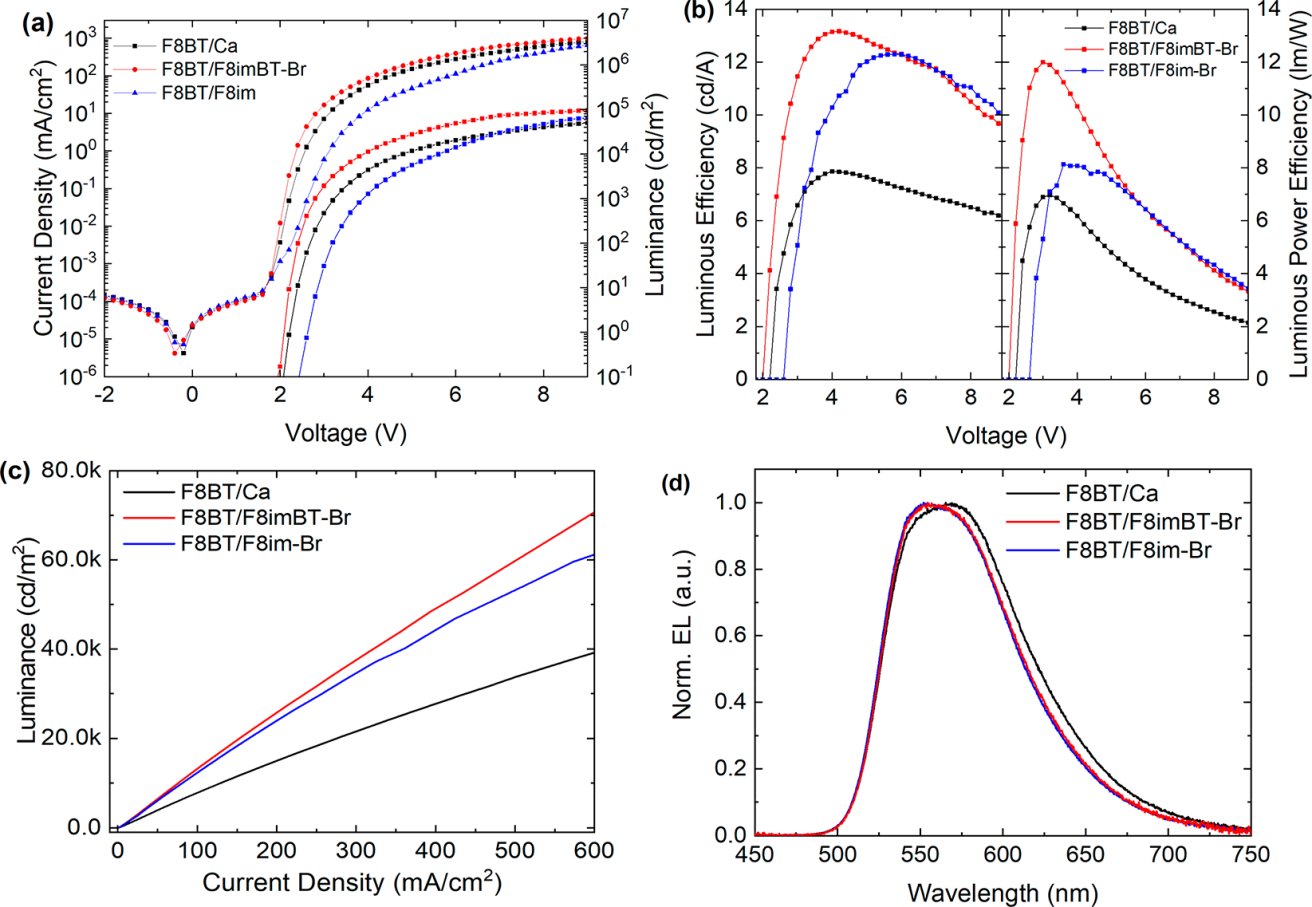
F8BT LEP device characteristics. (a) J–V–L data for PLEDs with Ca/Al (black), F8imBT-Br/Al (red), and F8im-Br/Al (blue) cathodes. (b) Luminous (cd/A) and luminous power (lm/W) efficiencies. (c) Luminance versus current density and (d) device EL spectra (at 7001, 17 070, and 2609 cd/m^2^ for Ca, F8im-Br, and F8imBT-Br EIL devices, respectively).

We now examine three different LEP PLEDs fabricated with F8imBT-Br, F8im-Br, and Ca EILs. First, F8BT PLED devices were tested. Figure 2 shows (a) J–V–L and (b) luminous and luminous power efficiencies for these devices.

The F8imBT-Br and Ca devices show a sharp increase in current density after 1.8 V (reaching 16.7 and 7.1 mA/cm^2^ at 3.0 V) while the F8im-Br displays a more gradual increase in current density, reaching only 0.60 mA/cm^2^ at 3.0 V. Consistent with this, the corresponding luminance turn-on voltages (defined as the voltage, *V*_on_, at which the luminance exceeds 0.1 cd/m^2^) are 2.0, 2.1, and 2.6 V. This indicates that while electron injection into F8BT from F8imBT-Br and Ca is ohmic, electron injection from F8im-Br appears injection limited (Δϕ = 0.7 eV, see SI).

The peak luminous and luminous power efficiencies are (i) 13.2 cd/A (at 14 040 cd/m^2^) and 12.0 lm/W (at 1922 cd/m^2^) for F8imBT-Br and (ii) 12.3 cd/A (at 11 740 cd/m^2^) and 8.1 lm/W (at 482 cd/m^2^) for F8im-Br (Figure 2b). The lower *V*_on_ and steeply rising emission for F8imBT-Br enhances the luminous power efficiency of the device. Both CPE devices display greater efficiencies than the Ca device, which has maximum luminous and luminous power efficiencies of 7.9 cd/A (at 4404 cd/m^2^) and 7.0 lm/W (at 901 cd/m^2^) due to the Ca causing image charge quenching of the luminescence and a better charge balance within the device.^14^ Single carrier devices show that F8imBT-Br has improved electron injection relative to F8im-Br, while both CPEs are good hole blocking materials (Figure S6). The EL spectra of both CPE devices (Figure 2d) are the same and indicate no emission from the CPE layer. The Ca device shows a slight enhancement in the low energy shoulder and broader emission width likely due to changes in weak microcavity interference effects.^50,51^

**Figure 3:**
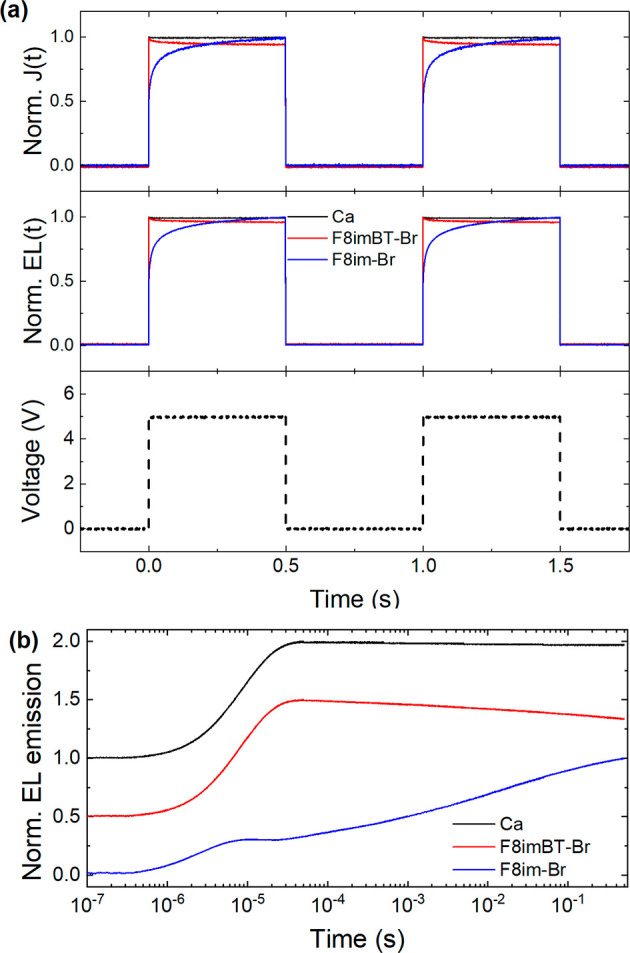
Transient response for F8BT LEP devices. (a) Normalized current density (top panel) and electroluminescence emission (middle panel) transients of F8BT PLEDs with Ca, F8imBT-Br, and F8im-Br EILs. The bottom panel shows the voltage pulse train used to excite each PLED. (b) Normalized EL transients plotted in a semilog plot. The curves are offset vertically for clarity.

To investigate the transient properties of these CPE PLEDs, the electroluminescence (EL(*t*)) and current density (*J*(*t*)) transients were recorded. The input signal used was a 1 Hz, 5 V square wave voltage train. The L and J transients for the Ca, F8imBT-Br, and F8im-Br F8BT devices are shown in Figure 3.

The reference Ca device shows a close to square wave response for both *J*(*t*) and EL(*t*), following the voltage transient, and has a rise time, *t*_r_, of 4.1 μs. The F8im-Br device, however, shows a slow rise for *J*(*t*) and EL(*t*) similar to previously reported CPE EIL PLED devices.^11,12,34^ The rise time, *t*_r_ (defined by the intersection between the tangent of the rising edge of the EL pulse and the saturation level of the EL, see Figure S7), of the electroluminescence transient is found to be on the order of ∼10^5^ μs, which is too slow for display applications (for the F8im-Br/Al device, the initial spike in the EL is disregarded for the purpose of determining the rise time).^15^ The initial rise in turn on is likely due to barrier-limited electron injection, while the subsequent slow rise in EL and *J* is due to a reduction of the injection barrier by ionic rearrangement within the F8im-Br. By comparison, the F8imBT-Br *J*(*t*) and EL(*t*) transients both show a fast rise time, *t*_r_ of 4.9 μs, followed by a minor decay in both the EL(*t*) and *J*(*t*) signals of ∼10% over the duration of the voltage pulse. This decrease is not, seemingly, a result of rapid degradation as the signal recovers to its original height for the second and subsequent voltage pulses. Normalized EL transient data were also measured for a F8BT/F8imBT-Br device at 5, 7, and 9 V (Figure S8) and very little change appears to occur for voltages within this range.

**Figure 4:**
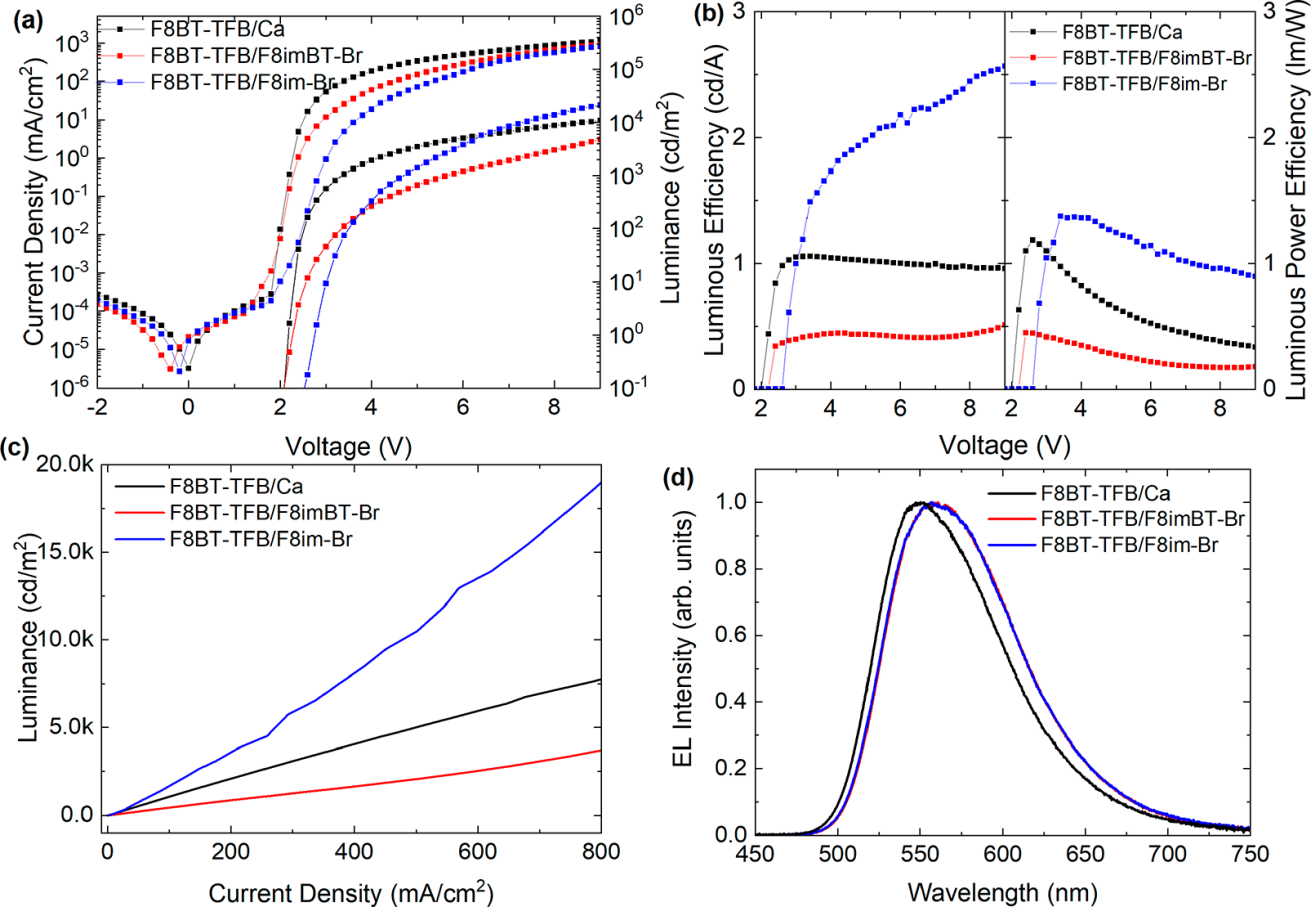
F8BT-TFB LEP device characteristics. (a) J–V–L data for PLEDs with Ca/Al (black), F8imBT-Br/Al (red), and F8im-Br/Al (blue) cathodes. (b) Luminous (cd/A) and luminous power (lm/W) efficiencies. (c) Luminance versus current density and (d) device EL spectra (at 3505, 662, and 1412 cd/m^2^ for Ca, F8im-Br, and F8imBT-Br EIL devices, respectively).

F8BT-TFB PLED devices were also tested with F8imBT-Br, F8im-Br, and Ca EILs. Figure 4a shows the J–V–L characteristics of these F8BT-TFB PLEDs, while Figure 4b shows the corresponding luminous and luminous power efficiencies.

Again, both Ca and F8imBT-Br EILs show a sharp luminance turn-on at *V*_on_ ∼ 2.1 V, while the F8im-Br CPE has a larger *V*_on_ = 2.6 V. As previously shown, Ca forms an ohmic electron injecting contact with F8BT,^52,53^ and the correspondence in turn-on voltage thus indicates that electron injection into the F8BT-TFB LEP is also ohmic for both the Ca/Al and F8imBT-Br/Al electrodes, while for F8im-Br/Al it is injection limited (Δϕ = 0.7 eV, see SI).

The peak luminous and luminous power efficiencies for the F8BT-TFB devices are (i) 2.6 cd/A (at 21 380 cd/m^2^) and 1.4 lm/W (at 74 cd/m^2^) for F8im-Br, (ii) 0.5 cd/A (at 4770 cd/m^2^) and 0.5 lm/W (at 12 cd/m^2^) for F8imBT-Br, and (iii) 1.1 cd/A (at 1074 cd/m^2^) and 1.2 lm/W (at 163 cd/m^2^) for Ca devices (Figure 4b). The EL spectra (Figure 4d) of the F8BT-TFB LEP devices only show emission from the LEP with no apparent CPE EIL emission, which would appear significantly to the blue, peaking at 429 nm for F8im-Br and 557 nm for F8imBT-Br (Figure S1b). The much weaker blue shift in EL emission for the Ca EIL reference devices is expected to be due to weak microcavity effects.^50,51^

Single-carrier devices were also fabricated (Figure S9) to confirm this explanation. Hole only devices show the hole blocking properties of both CPEs with lower current densities compared with Ca. For the electron only devices, the electron current density at 4 V is 3.5 times greater for F8imBT-Br than F8im-Br, confirming further that the former CPE does afford greater electron injection.

**Figure 5:**
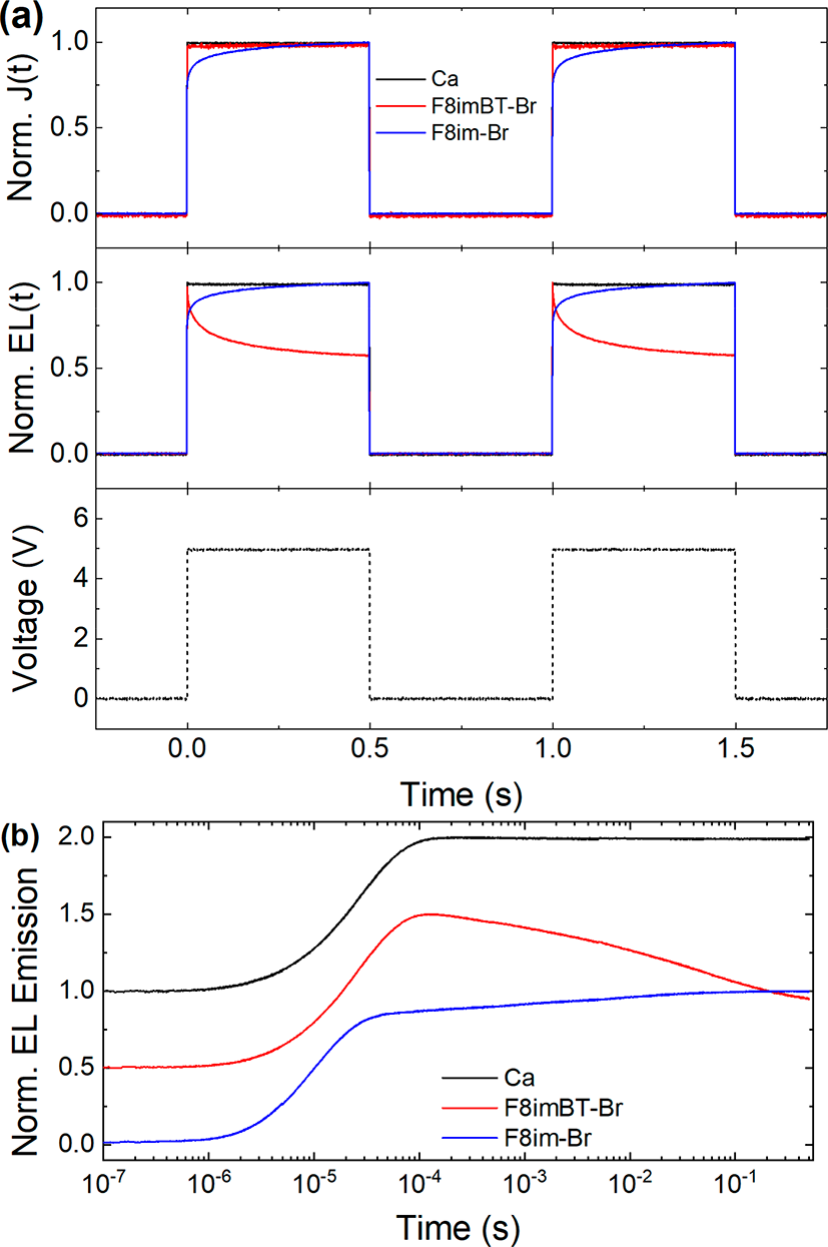
Transient response for F8BT-TFB LEP devices. (a) Normalized current density (top panel) and luminance (middle panel) transients of PLEDs with Ca, F8imBT-Br, and F8im-Br EILs. The bottom panel shows the voltage pulse train used to excite each PLED. (b) EL transients plotted in a semilog plot. The curves are offset vertically for clarity.

The luminance (EL(*t*)) and current density (*J*(*t*)) response transients for the F8BT-TFB LEP devices are shown in Figure 5a. Similar to the case for F8BT devices, the EL(*t*) and *J*(*t*) transients for the F8BT-TFB Ca reference devices follow closely the square wave voltage input signal. The F8im-Br device initially shows an equally fast rise (∼8.3 μs) before giving way to a more gradual rise out to ∼0.27 s. The latter slow rise is typical of CPEs containing mobile ions.^11,12^ However, the F8imBT-Br devices show a different EL(*t*) and *J*(*t*) response again. *J*(*t*) follows closely the square wave potential with a fast (∼7.9 μs) rise, as per the F8BT LEP case above. The EL(*t*) transient, in contrast, reaches an initial peak after ∼100 μs and then decays by 42% over the remaining 0.5 s pulse duration. Furthermore, as sequential EL(*t*) transients are unchanged (Figure 5a middle panel), this decay is evidently not due to irreversible degradation.

Additional EL(*t*) transients were measured for F8BT-TFB/F8imBT-Br devices for 1 Hz square wave pulses with different driving voltage amplitudes, ranging from 5 to 8 V (Figure S10). The data show that the decay in the EL(*t*) signal is faster when a larger driving voltage is used. For example, the decay is ∼42% when driven over a 0.5 s period at 5 V, while at 8 V the corresponding reduction is ∼55%.

**Figure 6:**
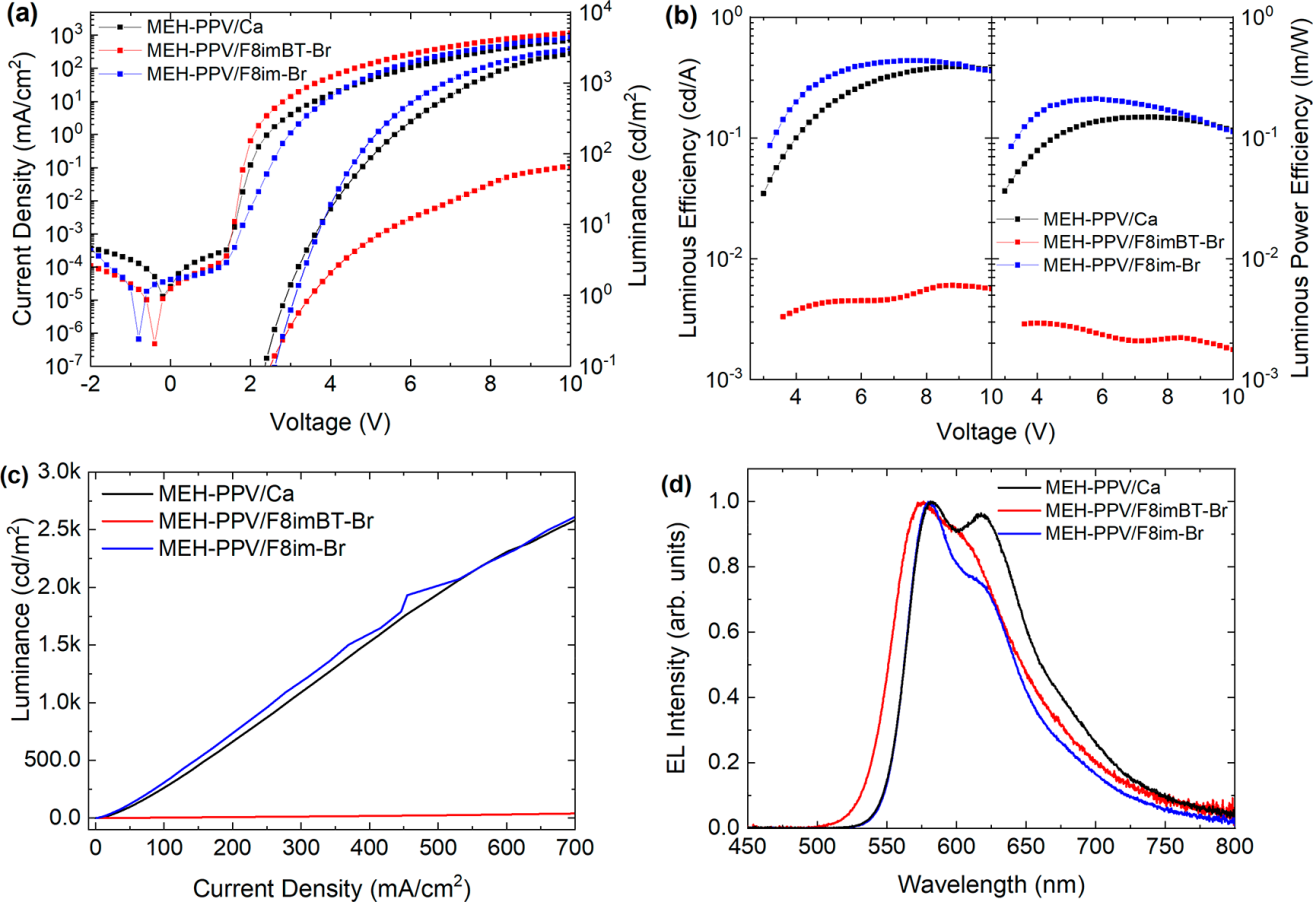
MEH-PPV LEP device characteristics. (a) J–V–L data for PLEDs with Ca/Al (black), F8imBT-Br/Al (red), and F8im-Br/Al (blue) cathodes. (b) Luminous (cd/A) and luminous power (lm/W) efficiencies. (c) Luminance versus current density and (d) peak normalized device EL spectra (at 1288, 1789, and 37.9 cd/m^2^ for Ca, F8im-Br, and F8imBT-Br EIL devices, respectively).

Last, devices were fabricated using MEH-PPV as LEPs with F8im-Br, F8imBT-Br, and Ca EILs. MEH-PPV and other dialkoxy substituted PPVs are known to be predominantly hole transporting polymers with poor electron transport.^47−49,54−57^ The strongly hole transporting nature of MEH-PPV means that, assuming adequate injection, the recombination zone will lie close to the MEH-PPV/CPE interface.^11^Figure 6 shows the resulting device characteristics.

Figure 6a shows the J–V–L data for the MEH-PPV PLEDs. As observed in the F8BT and F8BT-TFB devices, the sharp rise in current density indicates that the F8imBT-Br EIL facilitates good electron injection, while the F8im-Br devices are injection limited (Δϕ = 0.7 eV, see SI). We also studied single carrier device structures for MEH-PPV LEP devices, as for the F8BT-TFB LEP devices above. Figure S11a reports data for electron-only devices, confirming that F8imBT-Br delivers efficient electron injection into MEH-PPV. The current density for the F8imBT-Br device at 4 V is a factor of 6 greater than the F8im-Br devices and a factor of 2 relative to Ca devices. Figure S11b also shows that both F8imBT-Br and F8im-Br have good hole blocking properties consistent with the large offsets in their HOMO energies at 5.7 and 5.6 eV, respectively, relative to MEH-PPV at 5.1 eV.

Despite the facile electron injection that F8imBT-Br allows, the peak device efficiencies (0.006 cd/A (at 52.7 cd/m^2^) and 0.003 lm/W (at 2.7 cd/m^2^)) for corresponding MEH-PPV LEP devices are drastically reduced compared with both MEH-PPV/F8im-Br (0.44 cd/A (at 1503 cd/m^2^) and 0.22 lm/W (at 431 cd/m^2^)) and MEH-PPV/Ca (0.39 cd/A (at 1759 cd/m^2^) and 0.15 lm/W (at 1030 cd/m^2^); see Figure 6b).

Figure 6d shows the peak normalized EL spectra of the MEH-PPV PLEDs with Ca, F8im-Br, and F8imBT-Br EILs. The Ca and F8im-Br devices both show normal MEH-PPV emission with the main S_1_ to S_0_ 0–0 vibronic peak at 581 nm and the 0–1 vibronic peak at 618 nm and fwhm’s of 97 and 72 nm, respectively.^58,59^ The F8imBT-Br EIL device EL spectrum has an emission onset at a shorter wavelength (505 nm), with the main peak blue-shifted to 575 nm and a broader (compared to the F8im-Br device) fwhm of 98 nm. The spectral blue shift suggests the presence of emission from the F8imBT-Br CPE EIL in addition to that from MEH-PPV. This is supported by a comparison of the PL spectra of MEH-PPV, F8imBT-Br, and MEH-PPV/F8imBT-Br bilayer structures (Figure S12), with emission <530 nm arising from the F8imBT-Br. Voltage dependent EL measurements (Figure S13) also show that increasing the voltage increases the relative amount of F8imBT-Br emission present in the spectra. The loss in intensity in the red edge could be due to a microcavity effect from the shift in recombination zone toward the F8imBT-Br.

**Figure 7:**
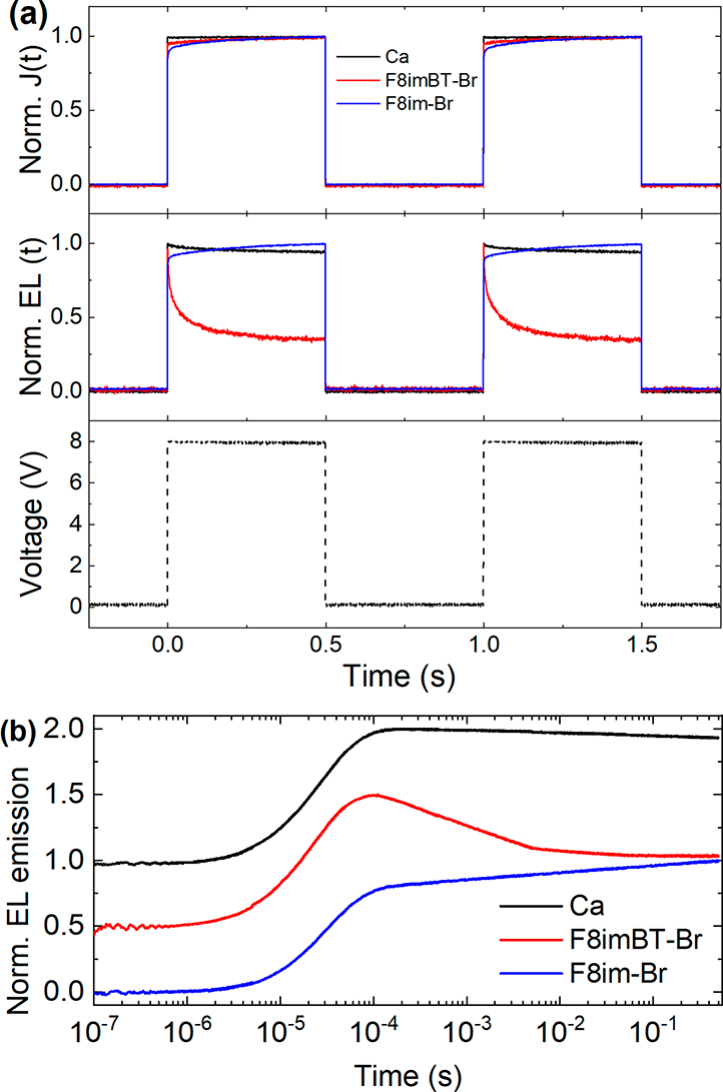
Transient response for MEH-PPV LEP devices. (a) Normalized current density (top panel) and luminance (middle panel) transients of PLEDs with Ca, F8imBT-Br, and F8im-Br EILs. The bottom panel shows the voltage pulse train used to excite each PLED. (b) EL transients plotted in a semilog plot. The curves are offset vertically for clarity.

Figure 7a shows the *J*(*t*) and EL(*t*) transients for MEH-PPV LEP/Ca, F8im-Br, and F8imBT-Br EIL devices. While the EL(*t*) transients for the Ca and F8im-Br devices relatively closely follow their *J*(*t*) transients, the F8imBT-Br transients are markedly different. Similar to the previous F8BT and F8BT-TFB devices, the EL(*t*) and *J*(*t*) transients for the latter PLEDs show rapid turn-on times (∼4 μs). Following this rapid rise, *J*(*t*) plateaus (in similar vein to *J*(*t*) for Ca devices) but EL(*t*) peaks at ∼4.0 μs and then falls, yielding a 65% reduction in signal over the pulse duration. Again, the F8im-Br devices initially show a fast rise (∼4.2 μs) before then following a more gradual increase across the remaining duration of the pulse (out to 0.5 s; Figure 7b). We note that the Ca metal EIL devices also show a decrease in luminance after a fast turn on, but in that case the magnitude of the effect is substantially less than for F8imBT-Br devices.

As with the F8BT and F8BT-TFB LEP devices, additional luminance transient measurements were performed for MEH-PPV/F8imBT-Br devices with driving voltages between 5 and 9 V (Figure S14). Similar to the F8BT-TFB case, there is a more rapid decay of the EL signal during the pulse with increasing pulse amplitude, from ∼45% at 5 V to ∼70% at 9 V. This correlates with the increase in F8imBT-Br emission shown in the EL spectra (Figure S13).

An overall summary of F8BT, F8BT-TFB, and MEH-PPV LEP device performance parameters can be found in Table 2.

**Table 2:** Comparison of Performance Parameters for F8BT, F8BT-TFB, and MEH-PPV LEP PLEDs with F8imBT-Br, F8im-Br, and Ca EILs

light emitting polymer	EIL	turn-on voltage *V*_on_ (V)^a^	luminance (cd/m^2^)^b^	peak luminous efficiency (cd/A)	peak power efficiency (lm/W)	peak EQE (%)	response time (μs)
F8BT	Ca	2.1	7450	7.9 @ 4.0 V	7.0 @ 3.2 V	2.4 @ 4.0 V	4.1
	F8imBT-Br	2.0	13 388	13.2 @ 4.2 V	12.0 @ 3.0 V	3.9 @ 4.2 V	4.9
	F8im-Br	2.4	11 088	12.3 @ 5.8 V	8.1 @ 3.6 V	3.6 @ 5.8 V	9.2 × 10^5^
F8BT-TFB	Ca	2.1	1072	1.1 @ 3.4 V	1.2 @ 2.6 V	0.31 @ 3.4 V	7.6
	F8imBT-Br	2.1	443	0.5 @ 4.2 V	0.5 @ 2.4 V	0.13 @ 4.2 V	7.9
	F8im-Br	2.6	2056	2.5 @ 9.0 V	1.4 @ 3.4 V	0.78 @ 9.0 V	2.7 × 10^5^
MEH-PPV	Ca	2.4	261.3	0.39 @ 9.0 V	0.15 @ 7.4 V	0.17 @ 9.0 V	3.5
	F8imBT-Br	2.5	4.94	0.006 @ 8.8 V	0.003 @ 4.0 V	0.0027 @ 8.8 V	4.0
	F8im-Br	2.6	306.5	0.44 @ 7.6 V	0.22 @ 5.8 V	0.20 @ 7.6 V	1.8 × 10^5^

a Voltage required to produce 0.1 cd/m^2^ luminance.

b Luminance values taken at 100 mA/cm^2^.

### Interfacial Energetics As a Key Factor for PLED Performance

We now discuss how the difference in F8imBT-Br and F8im-Br CPE EIL device performance across F8BT, F8BT-TFB, and MEH-PPV LEP devices can be explained by the nature of the LEP/CPE interface formed and the interfacial energetics and relative charge mobilities of the LEP materials.

**Figure 8:**
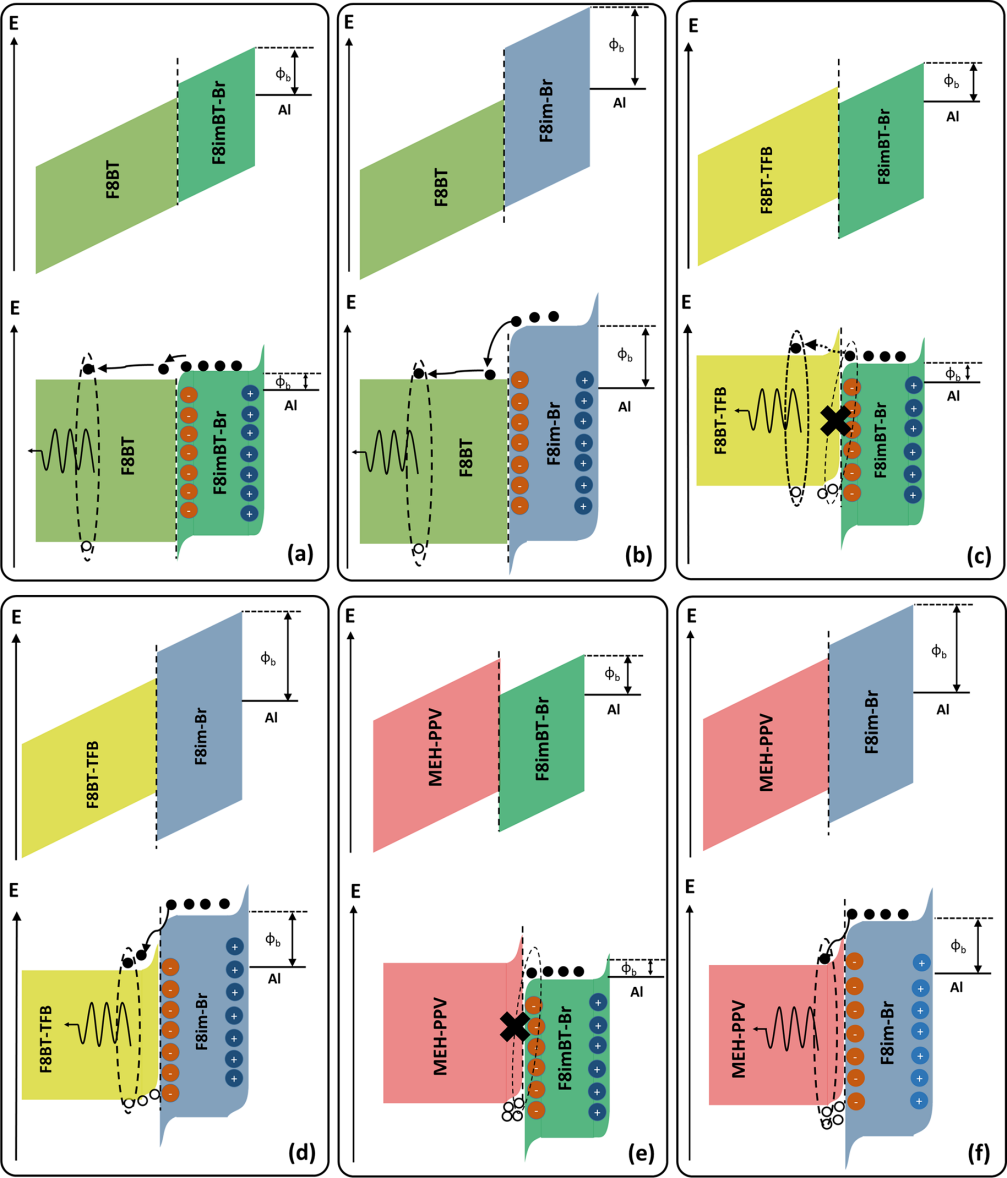
Schematic energy level diagrams of the interface between F8BT and (a) F8imBT-Br and (b) F8im-Br; F8BT-TFB and (c) F8imBT-Br and (d) F8im-Br; and MEH-PPV and (e) F8imBT-Br and (f) F8im-Br under forward bias in a PLED device. In each case, the upper diagrams show the interfaces that would be formed in the absence of CPE EIL ions and associated charge accumulation. The lower diagrams show the expected effects of ion redistribution and hole accumulation.

First, we note that all F8imBT-Br devices regardless of LEP show rapid luminance and current response times between 4.0 and 7.9 μs, similar to the Ca device response times (c.f. Table 2). This is likely due to the small electron injection barrier between Al and F8imBT-Br (∼0.1 eV, Figure S15) allowing for facile electron injection without needing substantial ionic rearrangement. While the F8imBT-Br eliminates the electron injection barrier from Al, using higher workfunction metals such as Ag and Au may introduce long device response times due to increased energetic barrier.^11^ By contrast, every F8im-Br device displays long luminance and current response times (∼10^5^ μs) as previously observed in wide band gap CPE based PLEDs.^11,12,34^ This can be explained by the electron injection barrier between Al and F8im-Br being much larger (∼0.7 eV– see Figure S15) and thus requires ionic rearrangement to form a tunnelling junction^5,11,23^ (Figure 8) for significant electron injection to be achieved. This highlights that a relatively deep LUMO is desirable from the point of view of device turn-on regardless of LEP used.

Second, we find that the efficiency of the device is strongly dependent on the type of interface formed between the LEP and the CPE. Using the F8im-Br CPE increases the luminous efficiency for each LEP compared to the control Ca device (likely due to a reduction in image charge quenching from the metal cathode and improved charge balance^15^); however, the F8imBT-Br CPE only produces an improvement in device efficiency for F8BT LEP devices with large reductions in device efficiency for F8BT-TFB (a factor of 2.4 and 6 compared to Ca and F8im-Br, respectively) and MEH-PPV (a factor of 63 and 74 compared to Ca and F8im-Br) LEP PLEDs. The factors driving this loss of efficiency are likely to be interfacial quenching due to (i) an increased LUMO energy level offset between F8imBT-Br and the LEP causing exciplex formation and nonradiative recombination, (ii) a smaller CPE energy gap reducing exciton confinement to within the LEP layer,^43^ and (iii) hole accumulation occurring at the interface causing the formation of quenching bipolaron states.^60−62^

We now turn our attention to differences in the F8imBT-Br PLED device efficiency for each LEP. In the case of F8BT, the F8imBT-Br shows the superior performance compared to Ca and F8im-Br, increasing EQE by a factor of 1.63 and 1.1, respectively. This is mainly due to improved charge carrier balance within the device due to facile electron injection from Al into the F8imBT-Br. No interfacial quenching would be expected to occur since the F8BT and F8imBT-Br energy levels are well aligned (Δ*E* of ∼0.1 eV), and electrons are efficiently transported away from the interface to radiatively recombine^40,63^ (Figure 8a).

This is not the case when using F8BT-TFB and MEH-PPV as the LEPs. When using F8BT-TFB as the LEP, the performance of the F8imBT-Br EIL device drops relative to that of the equivalent Ca and F8im-Br devices by a factor of 2.4 and 6, respectively. Using MEH-PPV as the LEP causes an even more severe relative decrease in efficiency for the F8imBT-Br device, with the EQE decreasing by a factor of 63 (compared to Ca) and 74 (compared to F8im-Br).

These cases differ with respect to F8BT in two ways; first, hole accumulation is expected to occur at both the F8BT-TFB/CPE interface (due to the hole transporting nature of the TFB units) and the MEH-PPV/CPE interface (which would not happen in F8BT devices due to the electron transporting nature of the material, which causes the recombination zone to be localized near the LEP/CPE interface. Second, there is a larger energy level offset between F8BT-TFB/F8imBT-Br (Δ*E*_HOMO_ = 0.2 eV and Δ*E*_LUMO_ = 0.2 eV) and MEH-PPV/F8imBT-Br (Δ*E*_HOMO_ = 0.6 eV and Δ*E*_LUMO_ = 0.4 eV) that is not present at the F8BT/F8imBT-Br interface.

Both of these phenomena are expected to lead to lower device efficiencies in the case of F8imBT-Br with F8BT-TFB and MEH-PPV. The hole accumulation leads to the formation of polarons and bipolarons (at higher charge density) for which the charges are coupled to localized lattice distortions in the LEP; this will lead to efficient exciton quenching without removing the charges.^60−62^ Meanwhile, the type II heterojunction energy level interfaces formed with F8BT-TFB/F8imBT-Br and MEH-PPV/F8imBT-Br lead to nonradiative recombination across the interface, which has been observed in previous polymer systems.^46^

Figure 8 shows the interfaces that are expected to form between each LEP and CPE. The upper diagrams in Figure 8 show the situation without taking account of either ion redistribution or hole accumulation, resulting in a type I heterojunction in the case of the F8im-Br EIL and type II for F8imBT-Br (with F8BT-TFB and MEH-PPV as the LEP). The bottom diagrams in Figure 8 show the expected changes following ionic redistribution and hole accumulation.^4,11^ Under applied bias, the ionic groups within the CPE should move to form an n-type region near the LEP/CPE interface and a p-type region near the CPE/Al interface,^11,64^ similar to the situation found in LECs.^65−67^ A significant voltage drop is then expected across these regions, leading to large band bending at the interfaces of the CPE.

The band bending due to both the ionic rearrangement and hole accumulation can offset the LUMO and HOMO levels further (Figure S16), causing increased interfacial luminance quenching. Evidence for this increase in quenching is provided from the luminance transients of F8BT-TFB/F8imBT-Br (Figure 5) and MEH-PPV/F8imBT-Br (Figure 7) devices, which both show large luminance decays over the course of ∼100 ms, which is consistent with the time scale of ionic rearrangement.^5,15^ As the ions rearrange due to the application of the electric field, interfacial band bending increases, leading to a greater energy offset, which in turns causes the observed drop in luminance due to increased interfacial quenching. Further evidence of this effect can be seen by increasing the size of the voltage pulse—this leads to a greater drop in the luminance transient of the aforementioned devices due to increased ionic rearrangement/hole accumulation (Figures S10 and S14).

We further note that the relative drop in performance for the MEH-PPV/F8imBT-Br device is much more severe here than for F8BT-TFB/F8imBT-Br devices, suggesting a greater influence from the CPE ions. This is likely due, at least in part, to a larger energy level offset (Δ*E* = 0.6 eV) between MEH-PPV and F8imBT-Br and a correspondingly greater hole accumulation at the interface, leading to the recombination zone being located close to an efficient exciton quenching site. The fact that EL emission is seen with the spectral characteristics of the EIL in the case of MEH-PPV/F8imBT-Br devices shows that some exciton transfer occurs across the interface.

Previous reports have explained this low device efficiency as poor electron injection/transport from F8BT-based CPEs into the LEP.^37^ However, our results show that, in fact, F8imBT-Br-based devices show facile electron injection into all LEPs; instead, we show that poor PLED device performance is caused by a combination of hole accumulation, energy level offsets, and ionic rearrangement causing luminescence quenching internally within the device rather than a lack of charge injection. Thus, future design strategies should focus on how to prevent these phenomena occurring within CPE based devices, while at the same time allowing fast (∼10^–6^ s) luminance turn-on times.

Table 3 summarizes the parameters tested and the phenomena that occurs.

**Table 3:** Summary of LEP/CPE Interface Properties and Expected Device Impacts

interface properties	expected influence on device performance	
LUMO energy offset	if *E*_CPE-LUMO_ ≤ *E*_LEP-LUMO_, then minimal quenching at the interface is expected; examples: F8im-Br/F8BT-TFB, F8im-Br/MEH-PPV, F8imBT-Br/F8BT	if *E*_CPE-LUMO_ > *E*_LEP-LUMO_, quenching is expected at the interface due to formation of nonradiative species across the interface; a larger LUMO energy level offsets leads to increased quenching; examples: F8imBT-Br/F8BT-TFB, F8imBT-Br/MEH-PPV
LEP charge transport properties	accumulation of holes at a type II interface locates the recombination zone closer to the CPE/LEP interface; bipolaron formation leads to efficient exciton-charge carrier quenching; hole tunnelling into the CPE layer can also further decrease efficiency through cross-interface exciplex formation and a corresponding increase in nonradiative decay	
	a critical consideration is the relative mobility of positive and negative charge carriers within the LEP; if the hole transport is strongly favored, the device efficiency suffers, independent of which of the three EILs is selected	
CPE energy gap	CPEs with a larger energy gap may reduce quenching by blocking the transfer of excitons from LEP to CPE^43^	
Al/CPE barrier	a deeper LUMO level allows for a smaller initial electron injection barrier from Al into CPE and accounts for fast current and luminance turn-on; shallower CPE LUMO levels lead to slower device turn-on times.	

## Conclusion

In the research reported here, we have explored in detail how the combination of LEP transport properties and the nature of the LEP/CPE interface determine PLED device performance for a range of different LEP and CPE combinations. We find that the formation of a type II heterojunction can lead to exciton quenching across the interface, with the relative PLED efficiency decreasing as the energy level offset between the CPE and LEP increases. This is observed when F8imBT-Br forms a type II heterojunction with all LEPs, however the energy level offset is minimal with F8BT (good device performance), intermediate with F8BT-TFB (poor device performance), and large with MEH-PPV (very poor device performance). This effect is exacerbated by hole accumulation at the LEP/CPE interface as well as ionic rearrangement within the CPE layer.

It is thus desirable to form a type I heterojunction (as observed with F8im-Br) which has good device performance across all LEPs, however the device turn-on times are slow (10^5^ μs) due to the shallow LUMO causing a large electron injection barrier with Al.

Important considerations emerge from these deductions that impact the design of CPE electron injection materials for PLEDs. To achieve both fast PLED response times and high device efficiency for all LEPs, a “universal” CPE should be designed in a way that it has a deep enough LUMO to provide ohmic electron injection from Al but shallow enough (and with a wide enough band gap) to prevent a large energy level offset (and subsequent luminescence quenching) with a wide range of LEPs. A possible route could be CPEs using F8-BT based backbones with minor (1–10%) fractions of the BT unit could be used to achieve an intermediate LUMO level between that of F8im-Br and F8imBT-Br or blending CPE materials with complementary characteristics.

An alternative approach could be the selection of LEPs that efficiently transport the injected electrons away from the interface to allow exciton formation within the bulk of the LEP or blending hole transporting with an electron transporting/hole blocking host to further tune the recombination zone location within the emissive layer.

## Experimental Section

F8imBT-Br and F8im-Br were synthesized according to previous reports.^15^ F8BT (*M*_n_ = 91 kDa), TFB (*M*_n_ = 60 kDa), and F8BT-TFB (*M*_n_ = 40 kDa) were sourced from Cambridge Display Technology Ltd. (CDT) and used as received. MEH-PPV (*M*_n_ = 70 kDa) was obtained from Sigma-Aldrich and used as received.

### UV–Vis and Photoluminescence Spectroscopy Measurements

Optical spectroscopy was performed on polymer thin films deposited on quartz. Quartz substrates were cleaned via sonication in acetone for 15 min, then IPA for 15 min, and finally 2% Hellmanex III in DI water solution for 15 min. Substrates were then plasma ashed in oxygen at 80 W power for 3 min using an Emitech K1050X. Films were spin-coated from solution onto quartz where settings were obtained to achieve ∼70 nm from toluene solution.

Absorption spectra were recorded using a UV–visible spectrophotometer (UV-2550, Shimadzu) and photoluminescence spectra using a spectrofluorometer (FluoroMax-3).

### PLED Fabrication

Patterned indium tin oxide (ITO)-on-glass substrates (size 12 mm × 8 mm) were cleaned in a succession of ultrasonic baths using acetone, isopropanol, and detergent (Hellmanex III, 2% by volume in DI water) for 15 min each, followed by oxygen plasma ashing in an Emitech K1050X. A 35 nm layer of PEDOT:PSS (Clevios P VP from Heraeus) was deposited by spin coating at 3000 rpm and then annealed in the air for 15 min at 135 °C followed by a 12 nm layer of TFB spin-coated from a 2 mg/mL toluene solution at 1000 rpm for 30 s. The TFB layer was annealed at 180 °C for 60 min. A 70 nm emissive layer of either the F8BT, F8BT-TFB random copolymer, or MEH-PPV was next deposited via spin coating from a 10 mg/mL solution in toluene at 2000 rpm, followed by a 10 nm layer of the CPE from a 2.5 mg/mL 2-methoxyethanol solution. Finally, a 100-nm-thick Al metal layer was thermally evaporated on top, without an intermediate annealing step, inside an MBraun glovebox evaporator (1 × 10^–6^ mbar).

### Device Characterization

PLEDs were characterized in an airtight sample chamber under nitrogen, which was connected to a Keithley SourceMeter controlled by a PC. The source meter applied a voltage to the chosen pixel and measured the resultant current, while a Minolta LS100 luminance meter recorded the pixel luminance. Electroluminescence spectra were measured using an Ocean Optics USB 2000 CCD spectrometer and were measured at 5 V for F8BT and F8BT-TFB devices and at 8 V for MEH-PPV devices.

The PLED EL intensity and current density transient responses were probed using an S-6 HP 3325B pulse generator and monitored with a digital oscilloscope (Tektronix DPO 3054); a fast Si-photodiode was used to detect the EL signal. All measurements were carried out within a nitrogen-filled test chamber.

## Supporting Information Available

UV–vis absorption and PL measurements for each LEP and CPE, APS measurement data for both CPEs and F8BT-TFB, single carrier device data, additional voltage dependent EL transient data, additional EL spectra for MEH-PPV/F8imBT-Br devices, and Kelvin probe workfunction measurements (PDF)
