# Supplementary material for: Optimizing Interfacial Energetics for Conjugated Polyelectrolyte Electron Injection Layers in High Efficiency and Fast Responding Polymer Light Emitting Diodes
Source: ACS Appl Mater Interfaces. 2022 May 18;14(21):24668–80. doi: 10.1021/acsami.2c05640 (PMC9164195; doi:10.1021/acsami.2c05640)
Supplement: Supplementary file 1 — am2c05640_si_001.pdf [file am2c05640_si_001.pdf]

Supporting Information

Optimising interfacial energetics for conjugated polyelectrolyte electron injection layers in high efficiency and fast responding polymer light emitting diodes

Iain Hamilton[†,‡] , Minwon Suh[†], Jim Bailey[†], Donal D. C. Bradley[‡] and Ji-Seon Kim[†]

[†] *Department of Physics and Centre for Processable Electronics, Imperial College London, London SW7 2AZ, UK*
[‡] *Division of Physical Sciences and Engineering, King Abdullah University of Science and Technology (KAUST), Thuwal, 23955-6900 Saudi Arabia*

UV-vis absorption of conjugated polyelectrolytes

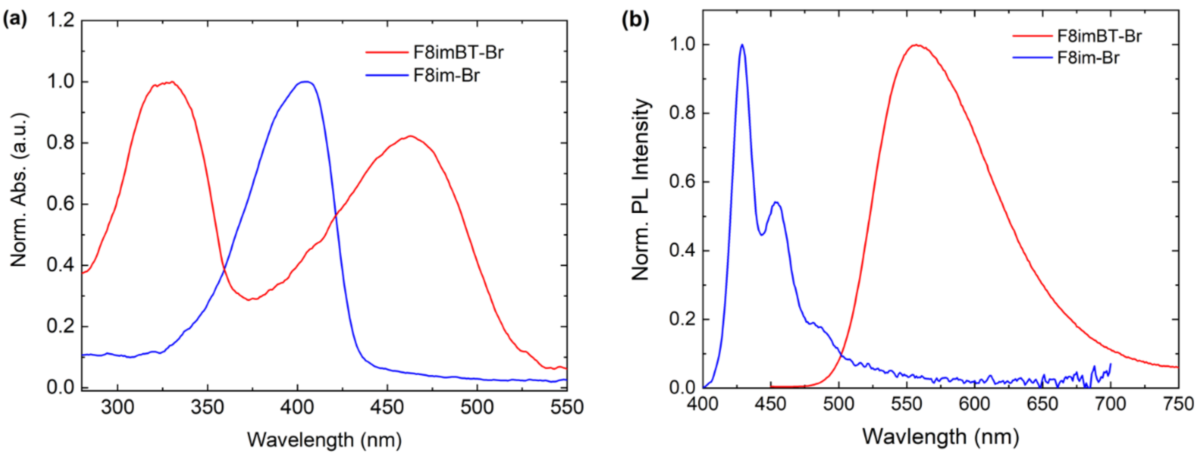

Figure S1: Thin film (20 nm) (a) UV-vis absorption and (b) photoluminescence spectra for F8im-Br and F8imBT-Br.

The F8im-Br absorption spectra shows only one broad absorption peak centred at ~404 nm and represents a π-π* HOMO-LUMO transition with both the HOMO and LUMO delocalised over the fluorene units. The absorption peak represents a red-shift of ~20 nm from the neutral form of F8im-Br (PFO[S1]), possibly indicating the fluorene chains adopt a more planar conformation compared to PFO.

The F8imBT-Br shows a similar absorption profile to F8BT (see **Figure S1(a)**) with a low energy absorption peak centred at ~463 nm, relating to the charge-transfer absorption band that arises from localisation of the LUMO on the BT unit.[S2] The higher energy absorption peak centred at ~330 nm relates to transitions from the HOMO to higher energy LUMOs that are delocalised across the polymer chain.[S3] In F8imBT-Br, the ratio of the low energy absorption intensity (I_{463}) to the high energy absorption intensity (I_{330}) is 0.82 – lower than for F8BT

(1.03). This indicates the ions could disrupt the degree of localisation of the LUMO on the BT unit, weakening the dipole oscillation strength of the transition.[S4,S5]

Air photoemission spectroscopy

To find the HOMO energy level of the F8im-Br and F8imBT-Br CPEs, air photoemission spectroscopy (APS) measurements were taken and the data shown below in **Figure S2**. F8imBT-Br and F8im-Br films were made by spin coating on ITO substrates. By extrapolating the linear fit of the cube root of the photoemission to baseline, we find the HOMO energy level for F8imBT-Br to be 5.73 eV, whilst the HOMO of F8im-Br is 5.58 eV. The HOMO energy levels of the F8BT, F8BT-TFB and MEH-PPV LEPs were also measured and found to be 5.85, 5.50 and 5.12 eV respectively (**Figure S3**).

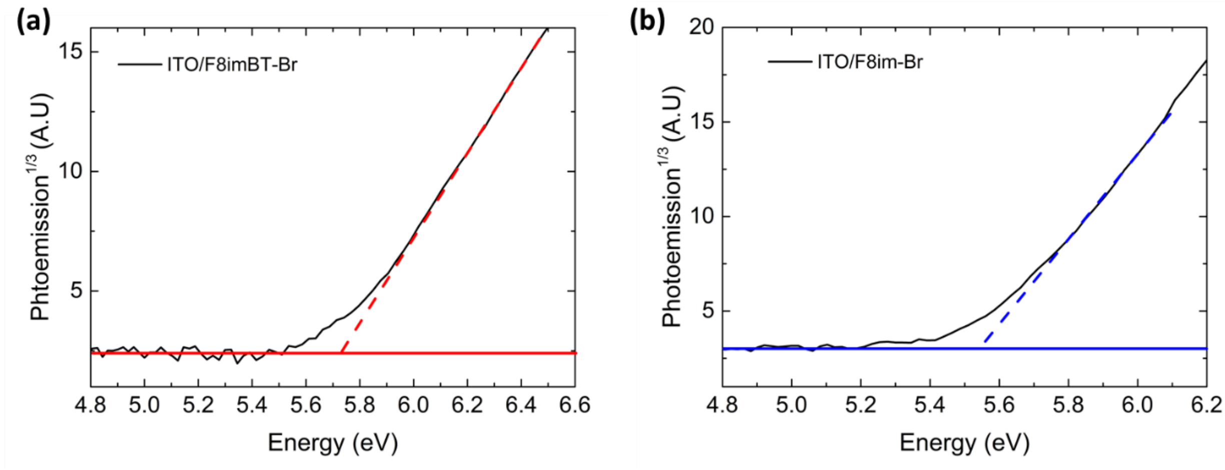

Figure S2: APS spectra for (a) F8imBT-Br and (b) F8im-Br.

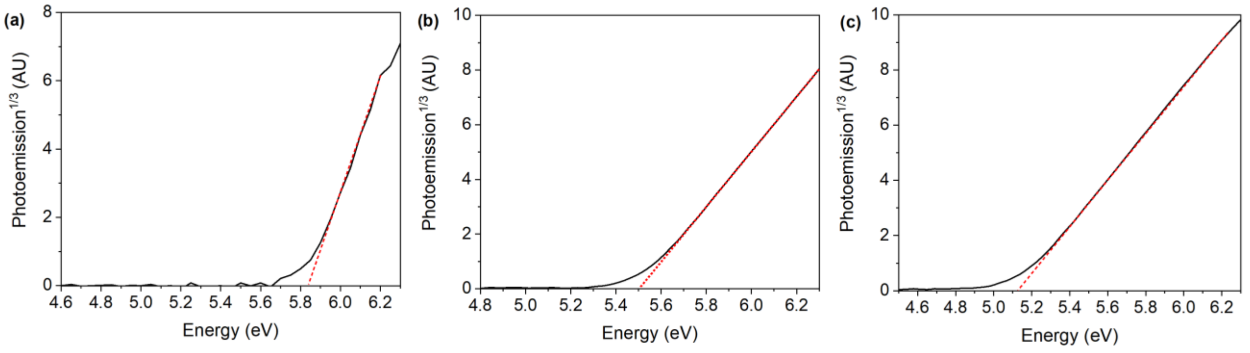

Figure S3: Air photoemission spectra for (a) F8BT, (b) F8BT-TFB random copolymer and (c) MEH-PPV. The extracted HOMO values was found to be 5.9, 5.5 and 5.1 eV respectively.

Table S1: Table showing values of HOMO, band gap and calculated LUMO.

Polymer	HOMO (eV)	Band Gap (eV)	LUMO (eV)
F8imBT-Br	5.73	2.36	3.37
F8im-Br	5.58	2.88	2.70
F8BT	5.85	2.40	3.45
F8BT-TFB	5.50	2.28	3.22
MEH-PPV	5.12	2.13	2.99

Characterisation of light emitting polymers

The first LEP examined is F8BT is well-known as an electron transporting copolymer[S6] which has relatively deep HOMO and LUMO energy levels (5.9 and 3.5 eV respectively) and a comparatively high electron mobility (up to ~5 × 10^{-3} cm^2/Vs[S7]) and relatively low hole mobility (10^{-8} to 10^{-6} cm^2/Vs[S8]). This imbalance leads to a RZ that is close to the anode at the LEP/interlayer (IL) interface[S9].

The second LEP tested is a statistical copolymer comprising F8BT and TFB units, referred to here as F8BT-TFB copolymer The inclusion of TFB units into the F8BT backbone has two effects, (i) shifting the HOMO to 5.5 eV (**Figure S3**) and (ii)the hole and electron currents become more balanced due to the hole transporting nature of the TFB unit.[S11,S12] The optical gap is slightly smaller compared to F8BT (2.3 eV – see **Figure S4**).

The third and final LEP studied is poly(2′-methoxy-5-2′-ethylhexyloxy)-1,4-phenylenevinylene) (MEH-PPV), which is a red-orange emitting polymer with relatively shallow energy levels of 5.1 and 3.0 eV for the HOMO and LUMO, respectively (**Figure S3**). This yields a smaller optical gap of 2.1 eV (**Figure S4**). As with most phenylene vinylenes (PPVs), it is a strongly hole dominated polymer (hole mobility, $\mu_h \approx 10^{-6}$ cm^2/Vs with electron mobility, $\mu_e < 10^{-8}$ cm^2/Vs)[S12–14] due to the presence of deep electron traps limiting electron transport.[S15–19] This has the effect of shifting the RZ close to the cathode at the LEP/CPE interface. This shift allows the role that the LEP/CPE interface plays in device performance to be more readily probed.

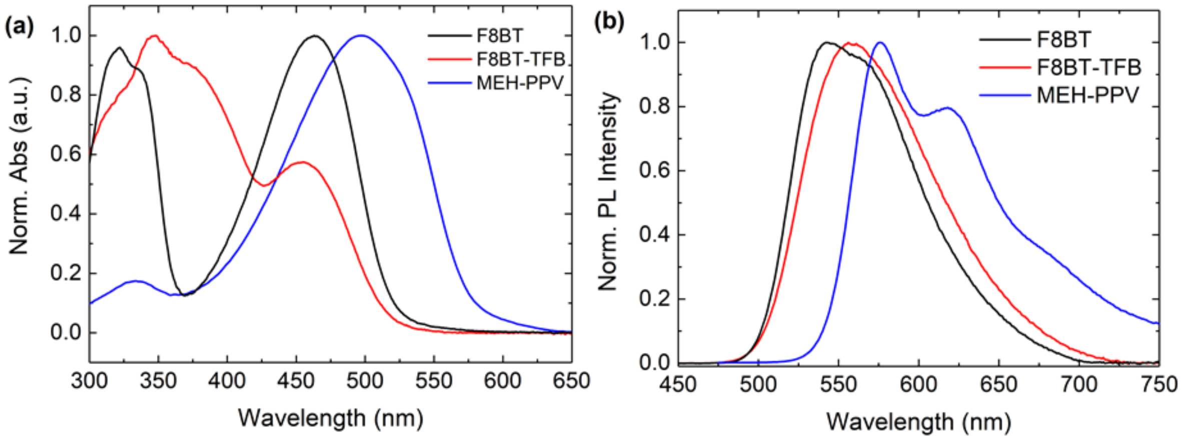

Figure S4: Plots showing (a) UV-vis absorption and (b) photoluminescence spectra for F8BT (black), F8BT-TFB (red) and MEH-PPV (blue) LEPs.

The localisation of the LUMO on the BT unit causes a 'charge-transfer' state to form which is the origin of the low energy absorption band at ~460 nm. The PL similarly shows a broad, featureless emission characteristic of charge transfer states.[S3]

The PL emission of F8BT-TFB (**Figure S4(b)**) shows only F8BT-like emission due to fast intra molecular energy transfer from TFB units to F8BT units, with a slightly red-shifted emission due to the enhanced charge transfer character in this copolymer.[S10]

The UV-vis absorption spectrum (**Figure S4(a)**) shows a broad absorption band at ~490 nm, whilst the PL spectrum (**Figure S4(b)**) shows a vibronic progression with the S_1-S_0 0-0 transition at ~570 nm. The relative strength of the vibronic peaks are strongly dependent on the morphology of the MEH-PPV layer.[S20–22]

AFM measurements

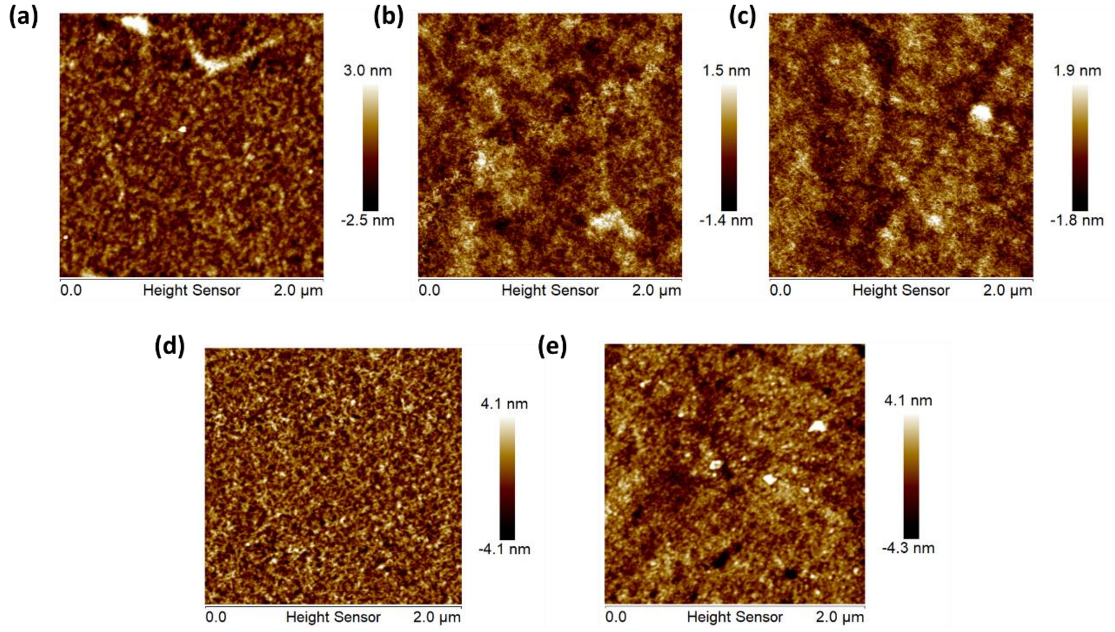

Figure S5: Atomic force microscopy measurements of (a) F8BT, (b) F8BT-TFB, (c) MEH-PPV, (d) F8im-Br and (e) F8imBT-Br.

Additional device characterisation

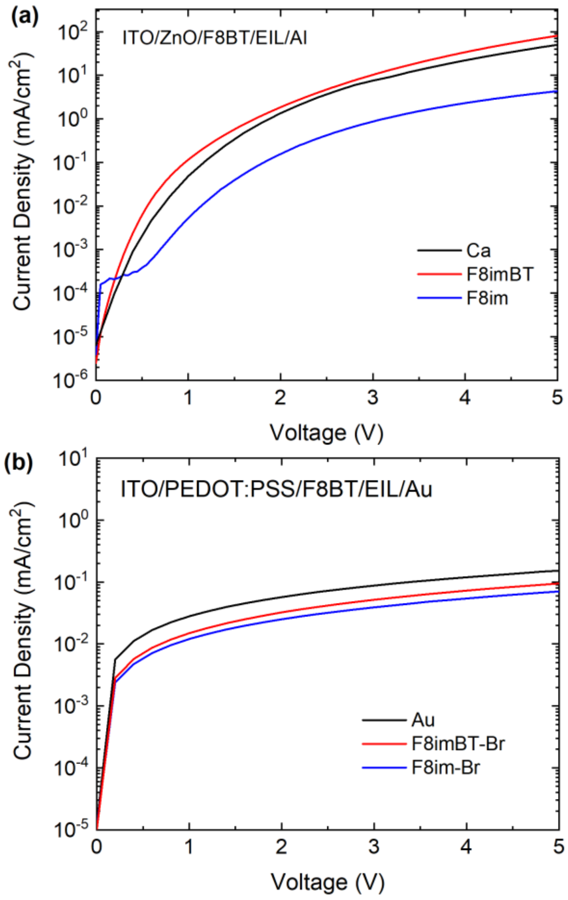

Figure S6: J-V measurements for (a) electron only and (b) hole only single carrier devices containing no CPE (black), F8imBT-Br (red) and F8im-Br (blue).

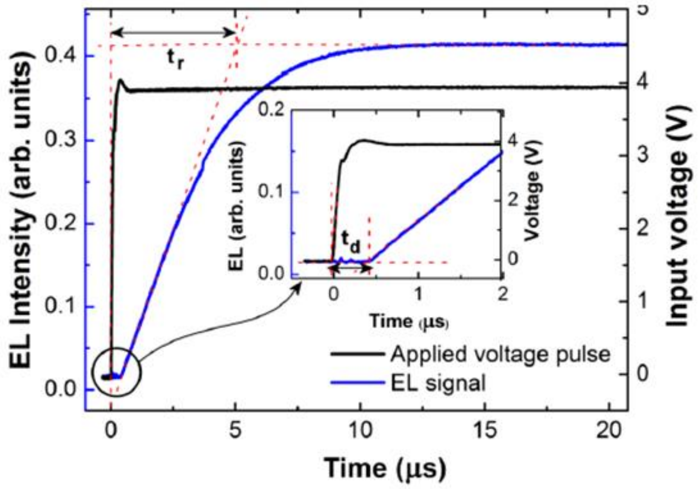

Figure S7: Plot showing applied pulse voltage (black) and EL intensity (blue) as a function of time. The distance between the rising edge of the voltage pulse and the rise of the EL signal (shown

clearly in inset) represent the delay time, t_d, whilst the intersection of the tangent of the rising edge of the EL pulse to the saturation level of the EL represents the rise time, t_r.

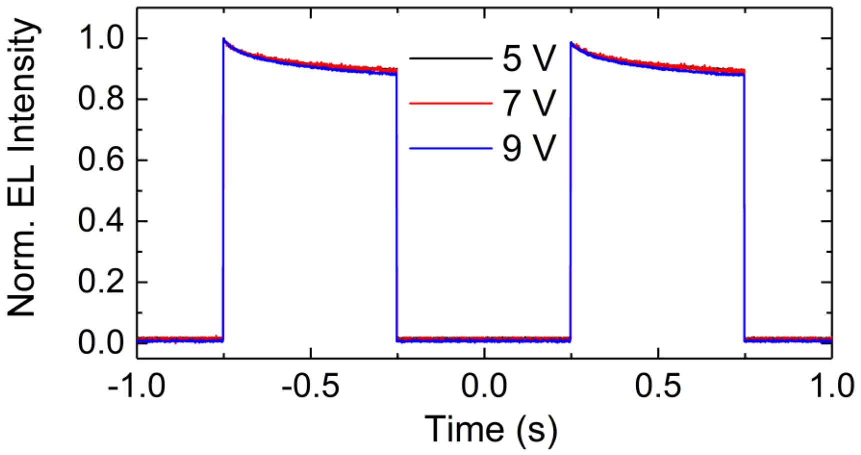

Figure S8: Normalised luminance transient of F8BT/F8imBT-Br devices taken at 5, 7 and 9 V, 1 Hz square wave voltage pulses.

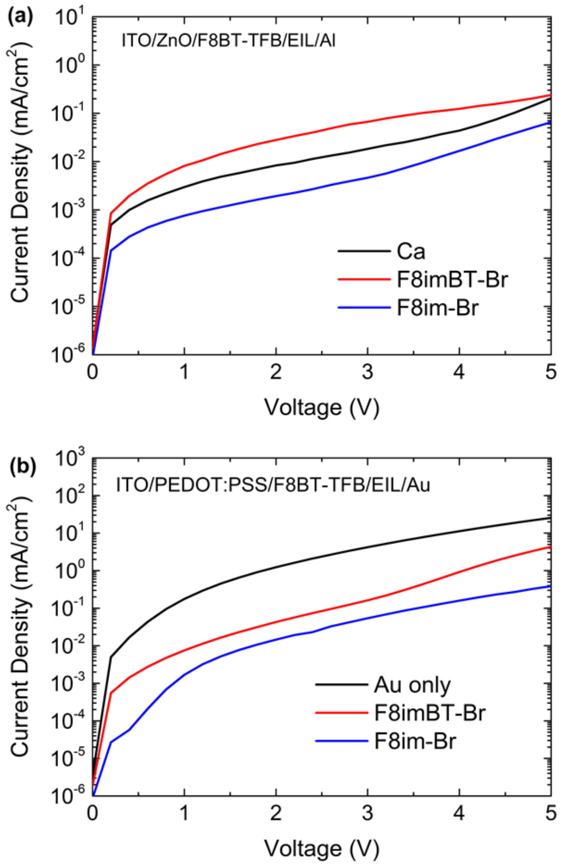

Figure S9: J-V measurements for (a) electron only and (b) hole only single carrier F8BT-TFB devices containing no CPE (black), F8imBT-Br (red) and F8im-Br (blue).

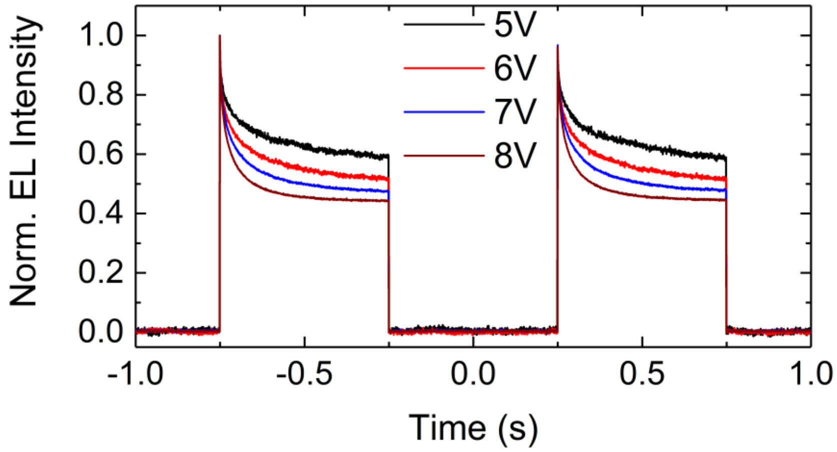

Figure S10: Normalised electroluminescence transients for F8BT-TFB/F8imBT-Br device with 5-8 V, 1 Hz square wave driving potentials.

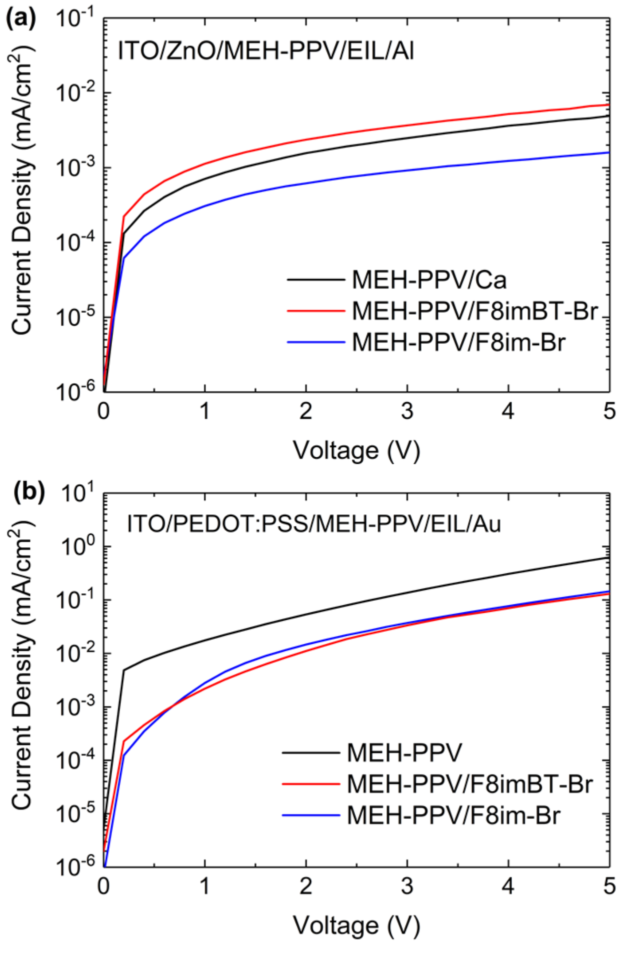

Figure S11: J-V measurements for (a) electron only and (b) hole only single carrier MEH-PPV devices containing no CPE (black), F8imBT-Br (red) and F8im-Br (blue).

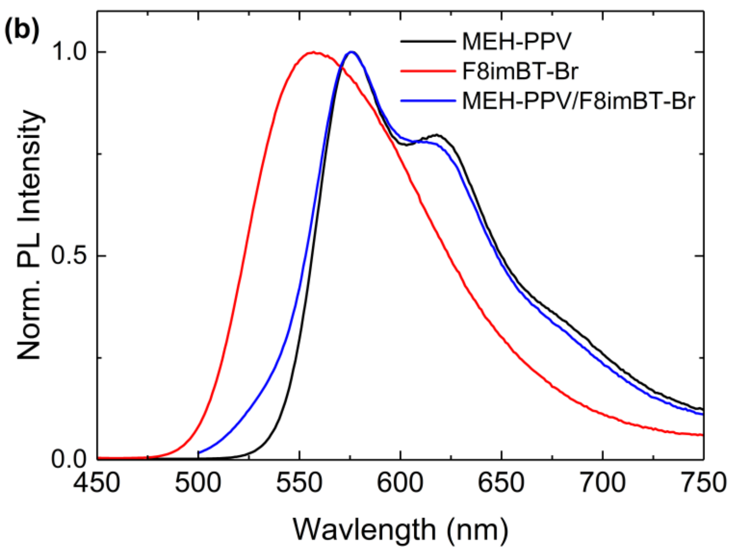

Figure S12: Normalised PL spectra for MEH-PPV (black), F8imBT-Br (red) and MEH-PPV/F8imBT-Br bilayer (blue).

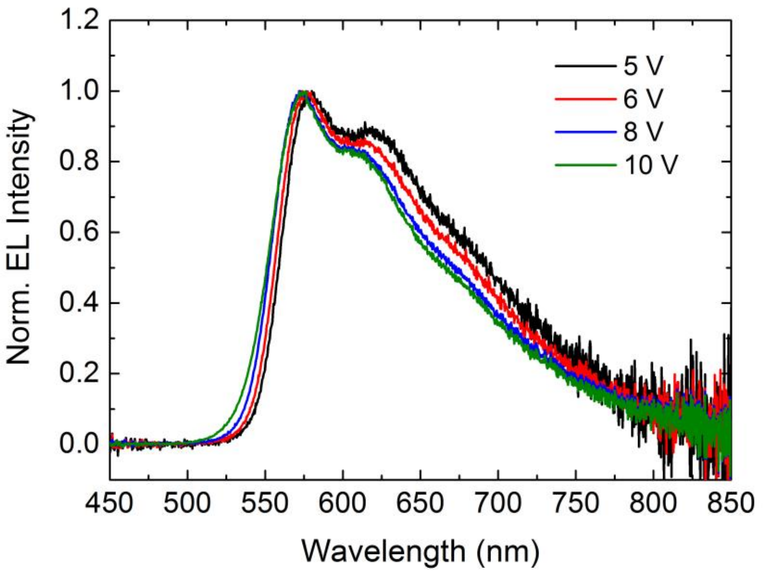

Figure S13: Normalised EL spectra of MEH-PPV/F8imBT-Br PLED devices driven at 5, 6, 8 and 10 V.

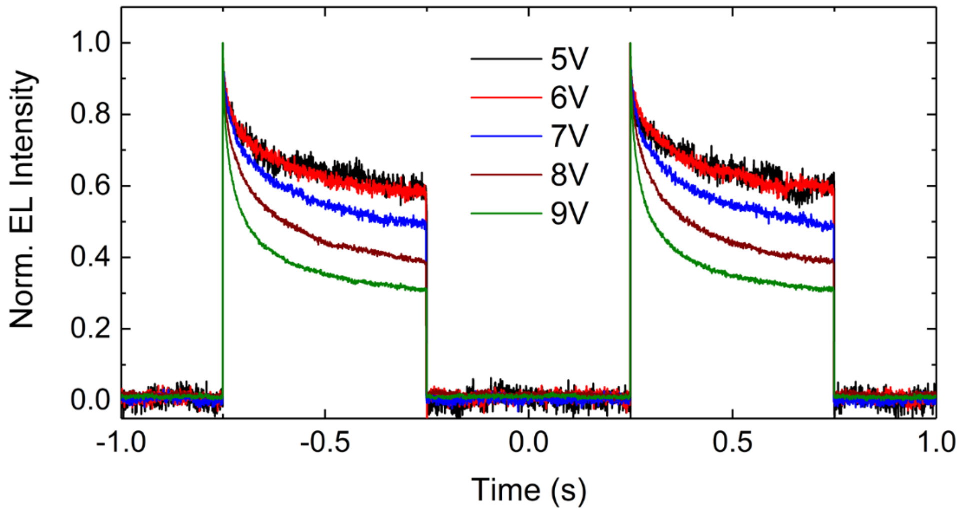

Figure S14: Normalised electroluminescence transients for MEH-PPV/F8imBT-Br device with 5-8 V, 1 Hz square wave driving potentials.

Kelvin Probe measurements

The Kelvin probe is an effective tool for probing the vacuum level shift at the metal/CPE interface, i.e. how much the workfunction of the Al cathode is shifted by the ionic dipoles of the CPE.[S10,S11] To identify the factors yielding fast turn on for F8imBT-Br based CPE devices, kelvin probe measurements were taken for bare Al, Al/F8imBT-Br (10 nm) and Al/F8im-Br (10 nm) samples (**Figure S15** below).

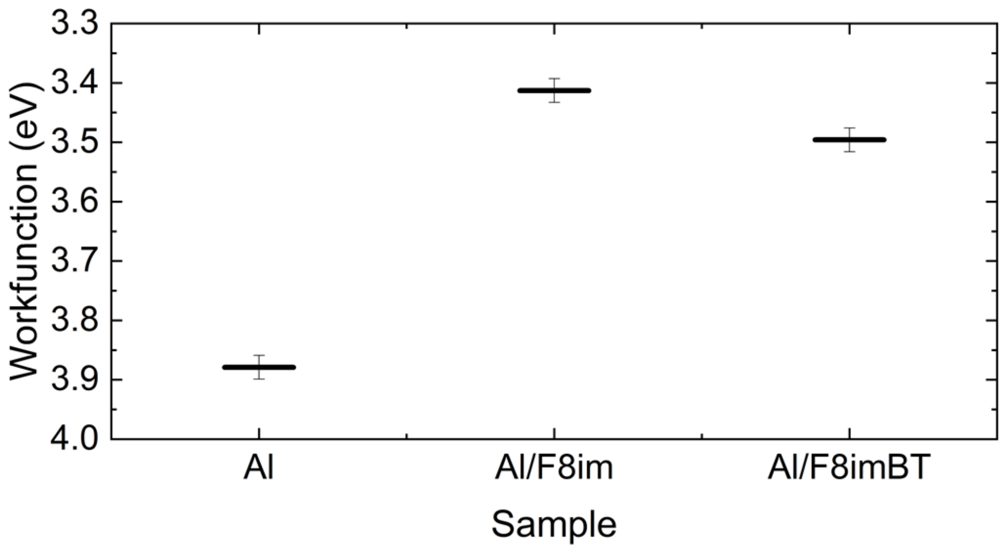

Figure S15: Workfunction measurements of Al, Al/F8im-Br and Al/F8imBT-Br samples.

The workfunction of the bare aluminium was found to be 3.88 eV, similar to the values reported (measured by the Kelvin probe technique in air) in the literature.[S23] The workfunction of the Al/F8im-Br sample was measured as 3.41 eV, a reduction in workfunction of 0.47 eV compared to Al. The Al/F8imBT-Br workfunction was measured as 3.50 eV, a reduction of 0.38 eV. Since the LUMO of F8imBT-Br is 3.37 eV, this allows ohmic electron injection to

occur from Al to F8imBT-Br as the barrier to electron injection is then only ~0.1 eV. The electron injection from Al to F8im-Br however remains barrier limited. The higher lying LUMO of F8im-Br (2.70 eV) means the electron injection barrier is much larger at ~0.7 eV. Using metal cathodes with deeper workfunctions (i.e. Ag or Au) is likely to increase the energy barrier from the cathode to the CPE, resulting in reduced electron injection and longer device response times.[S25]

MEH-PPV/F8imBT-Br interface energetics

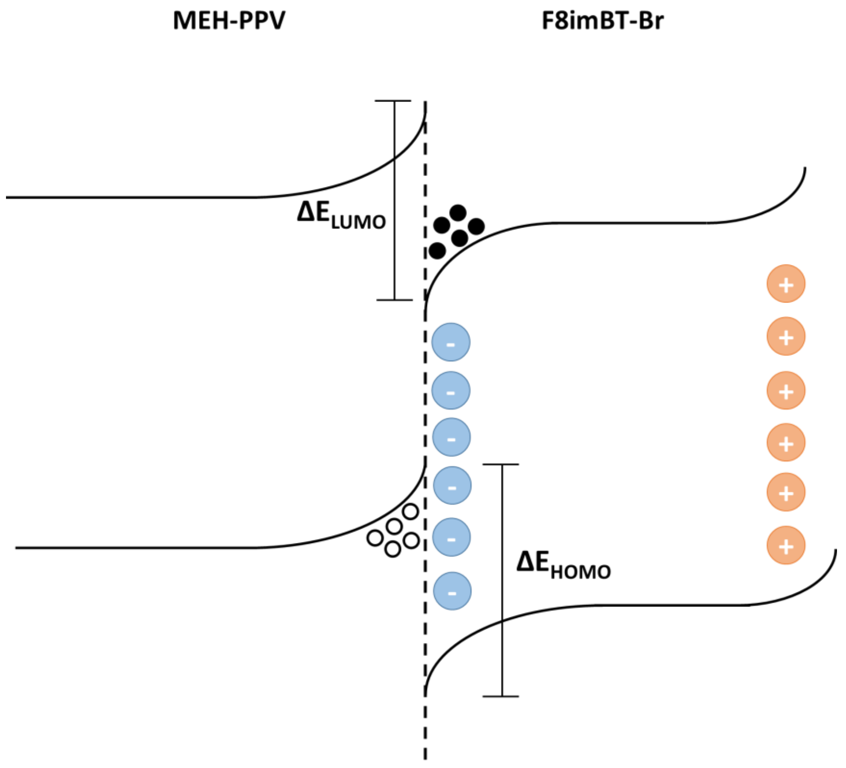

Figure S16: Schematic energy level diagram of the interface between MEH-PPV and F8imBT-Br. Ion redistribution in F8imBT-Br and hole accumulation in MEH-PPV causes band bending and hence a greater energy level offset possibly leading to increased non-radiative recombination.

References

(S1) Hamilton, I.; Chander, N.; Cheetham, N. J.; Suh, M.; Dyson, M.; Wang, X.; Stavrinou, P. N.; Cass, M.; Bradley, D. D. C.; Kim, J. S. Controlling Molecular Conformation for Highly Efficient and Stable Deep-Blue Copolymer Light-Emitting Diodes. *ACS Appl. Mater. Interfaces* **2018**, *10* (13), 11070–11082. https://doi.org/10.1021/acsami.8b00243.

(S2) Jespersen, K. G.; Beenken, W. J. D.; Zaushitsyn, Y.; Yartsev, A.; Andersson, M.; Pullerits, T.; Sundström, V. The Electronic States of Polyfluorene Copolymers with Alternating Donor-Acceptor Units. *J. Chem. Phys.* **2004**, *121* (24), 12613–12617. https://doi.org/10.1063/1.1817873.

(S3) Winfield, J. M.; Van Vooren, A.; Park, M.-J.; Hwang, D.-H.; Cornil, J.; Kim, J.-S.; Friend, R. H. Charge-Transfer Character of Excitons in Poly[2,7-(9,9-Di-n-Octylfluorene)(1-x)-Co-4,7-(2,1,3-Benzothiadiazole)(X)]. *J. Chem. Phys.* **2009**, *131* (2009), 035104. https://doi.org/10.1063/1.3177327.

(S4) Belton, C. R.; Kanibolotsky, A. L.; Kirkpatrick, J.; Orofi, C.; Elmasly, S. E. T.; Stavrinou, P. N.; Skabara, P. J.; Bradley, D. D. C. Location, Location, Location - Strategic Positioning of 2,1,3-Benzothiadiazole Units within Trigonal Quaterfluorene-Truxene Star-Shaped Structures. *Adv. Funct. Mater.* **2013**, *23*, 2792–2804. https://doi.org/10.1002/adfm.201202644.

(S5) Hodgkiss, J. M.; Tu, G.; Albert-Seifried, S.; Huck, W. T. S.; Friend, R. H. Ion-Induced Formation of Charge-Transfer States in Conjugated Polyelectrolytes. *J. Am. Chem. Soc.* **2009**, *131* (25), 8913–8921. https://doi.org/10.1021/ja902167a.

(S6) Campbell, A. J.; Bradley, D. D. C.; Antoniadis, H. Dispersive Electron Transport in an Electroluminescent Polyfluorene Copolymer Measured by the Current Integration Time-of-Flight Method. *Appl. Phys. Lett.* **2001**, *79* (14), 2133. https://doi.org/10.1063/1.1406143.

(S7) Donley, C.; Zaumseil, J.; Andreasen, J. W.; Nielsen, M. M.; Sirringhaus, H.; Friend, R. H.; Kim, J.-S. Effects of Packing Structure on the Optoelectronic and Charge Transport Properties in Poly (9, 9-Di-n-Octylfluorene-Alt-Benzothiadiazole). *J. Am. Chem. Soc.* **2005**, *127* (37), 12890–12899.

(S8) Blakesley, J. C.; Castro, F. A.; Kylberg, W.; Dibb, G. F. A.; Arantes, C.; Valaski, R.; Cremona, M.; Kim, J. S.; Kim, J. S. Towards Reliable Charge-Mobility Benchmark Measurements for Organic Semiconductors. *Org. Electron.* **2014**, *15* (6), 1263–1272. https://doi.org/10.1016/j.orgel.2014.02.008.

(S9) Bailey, J.; Wright, E. N.; Wang, X.; Walker, A. B.; Bradley, D. D. C.; Kim, J.-S. Understanding the Role of Ultra-Thin Polymeric Interlayers in Improving Efficiency of Polymer Light Emitting Diodes. *J. Appl. Phys.* **2014**, *115* (20), 204508. https://doi.org/10.1063/1.4879455.

(S10) Kim, J.-S.; Lu, L.; Sreearunothai, P.; Seeley, A.; Yim, K.-H.; Petrozza, A.; Murphy, C. E.; Beljonne, D.; Cornil, J.; Friend, R. H. Optoelectronic and Charge Transport Properties at Organic-Organic Semiconductor Interfaces: Comparison between Polyfluorene-Based Polymer Blend and Copolymer. *J. Am. Chem. Soc.* **2008**, *130* (39), 13120–13131. https://doi.org/10.1021/ja803766j.

(S11) Redecker, M.; Bradley, D. D. C.; Inbasekaran, M.; Wu, W. W.; Woo, E. P. High Mobility Hole Transport Fluorene-Triarylamine Copolymers. *Adv. Mater.* **1999**, *11* (3), 241–246. https://doi.org/10.1002/(SICI)1521-4095(199903)11:3<241::AID-ADMA241>3.0.CO;2-J.

(S12) Amorim, C. A.; Cavallari, M. R.; Santos, G.; Fonseca, F. J.; Andrade, a. M.; Mergulhão, S. Determination of Carrier Mobility in MEH-PPV Thin-Films by Stationary and Transient Current Techniques. *J. Non. Cryst. Solids* **2012**, *358* (3), 484–491. https://doi.org/10.1016/j.jnoncrysol.2011.11.001.

(S13) Bozano, L.; Carter, S. A.; Scott, J. C.; Malliaras, G. G.; Brock, P. J. Temperature- and Field-Dependent Electron and Hole Mobilities in Polymer Light-Emitting Diodes. *Appl. Phys. Lett.* **1999**, *74* (8), 1132–1134. https://doi.org/10.1063/1.123959.

(S14) Blom, P. W. M.; De Jong, M. J. M.; Vleggaar, J. J. M. Electron and Hole Transport in Poly(p-Phenylene Vinylene) Devices. *Appl. Phys. Lett.* **1996**, *68* (23), 3308–3310. https://doi.org/10.1063/1.116583.

(S15) Giebeler, C.; Whitelegg, S. A.; Campbell, A. J.; Liess, M.; Martin, S. J.; Lane, P. A.; Bradley, D. D. C.; Webster, G.; Burn, P. L. Optical Studies of Electric Fields in Poly(2-Methoxy-5-Ethyl(2(')- Hexyloxy) Para-Phenylene Vinylene) Light-Emitting Diodes. *Appl Phys Lett* **1999**, *74* (24), 3714–3716. https://doi.org/10.1063/1.123238.

(S16) Tuladhar, S. M.; Sims, M.; Kirkpatrick, J.; Maher, R. C.; Chatten, A. J.; Bradley, D. D. C.; Nelson, J.; Etchegoin, P. G.; Nielsen, C. B.; Massiot, P.; George, W. N.; Steinke, J. H. G. Influence of Alkyl Chain Length on Charge Transport in Symmetrically Substituted Poly(2,5-Dialkoxy-p-Phenylenevinylene) Polymers. *Phys. Rev. B* **2009**, *79* (3), 035201. https://doi.org/10.1103/PhysRevB.79.035201.

(S17) Zhang, Y.; De Boer, B.; Blom, P. W. M. Trap-Free Electron Transport in Poly(p-Phenylene Vinylene) by Deactivation of Traps with n -Type Doping. *Phys. Rev. B* **2010**, *81* (8), 1–5. https://doi.org/10.1103/PhysRevB.81.085201.

(S18) Campbell, A. J.; Bradley, D. D. C.; Lidzey, D. G. Space-Charge Limited Conduction with Traps in Poly(Phenylene Vinylene) Light Emitting Diodes. *J. Appl. Phys.* **1997**, *82* (12), 6326–6342. https://doi.org/10.1063/1.366523.

(S19) Craciun, N. I.; Zhang, Y.; Palmaerts, A.; Nicolai, H. T.; Kuik, M.; Kist, R. J. P.; Wetzelaer, G. A. H.; Wildeman, J.; Vandenbergh, J.; Lutsen, L.; Vanderzande, D.; Blom, P. W. M. Hysteresis-Free Electron Currents in Poly(p-Phenylene Vinylene) Derivatives. *J. Appl. Phys.* **2010**, *107* (12), 124504. https://doi.org/10.1063/1.3432744.

(S20) Nguyen, T.-Q.; Martini, I. B.; Liu, J.; Schwartz, B. J. Controlling Interchain Interactions in Conjugated Polymers: The Effects of Chain Morphology on Exciton−Exciton Annihilation and Aggregation in MEH−PPV Films. *J. Phys. Chem. B* **2000**, *104* (2), 237–255. https://doi.org/10.1021/jp993190c.

(S21) Iyengar, N. A.; Harrison, B.; Duran, R. S.; Schanze, K. S.; Reynolds, J. R. Morphology Evolution in Nanoscale Light-Emitting Domains in MEH-PPV/PMMA Blends. *Macromolecules* **2003**, *36* (24), 8978–8985. https://doi.org/10.1021/ma034908w.

(S22) Cossiello, R. F.; Akcelrud, L.; Atvars, T. D. Z. Solvent and Molecular Weight Effects on Fluorescence Emission of MEH-PPV. *J. Braz. Chem. Soc.* **2005**, *16* (1), 74–86. https://doi.org/10.1590/S0103-50532005000100012.

(S23) Oh, S. H.; Na, S. I.; Jo, J.; Lim, B.; Vak, D.; Kim, D. Y. Water-Soluble Polyfluorenes as an Interfacial Layer Leading to Cathode-Independent High Performance of Organic Solar Cells. *Adv. Funct. Mater.* **2010**, *20* (12), 1977–1983. https://doi.org/10.1002/adfm.200902386.

(S24) Hu, Z.; Zhang, K.; Huang, F.; Cao, Y. Water/Alcohol Soluble Conjugated Polymers for the Interface Engineering of Highly Efficient Polymer Light-Emitting Diodes and Polymer Solar Cells. *Chem. Commun.* **2015**, *51* (26), 5572–5585. https://doi.org/10.1039/C4CC09433F.

(S25) Hoven, C. V; Yang, R.; Garcia, A.; Crockett, V.; Heeger, A. J.; Bazan, G. C.; Nguyen, T.-Q. Electron Injection into Organic Semiconductor Devices from High Work Function Cathodes. *Proc. Natl. Acad. Sci. U. S. A.* **2008**, *105* (35), 12730–12735. https://doi.org/10.1073/pnas.0806494105.
